# Short- and long-term effects of emotion up- and down-regulation

**DOI:** 10.1162/imag_a_00028

**Published:** 2023-11-09

**Authors:** Kersten Diers, Anne Gärtner, Sabine Schönfeld, Denise Dörfel, Henrik Walter, Burkhard Brocke, Alexander Strobel

**Affiliations:** Faculty of Psychology, Technische Universität Dresden, Dresden, Germany; Evangelische Hochschule Dresden, Dresden, Germany; Division of Mind and Brain Research, Department of Psychiatry and Psychotherapy, CCM, Charité Universitätsmedizin, Berlin, Germany

**Keywords:** emotion regulation, amygdala, up-regulation, reappraisal, distancing, replication

## Abstract

It is an open question in cognitive emotion regulation research how emotion regulation unfolds over time, and whether the brain regions involved in down-regulation are also recruited during up-regulation of emotions. As a replication and extension of our preceding study, we conducted an fMRI study in young healthy adults on the neural basis of up- and down-regulation of negative and neutral pictures during the immediate stimulation phase as well as after short- and long-term delays (N=47 for immediate and short-term delays, a subset of N=30 for long-term delays). For this, we employed three experimental conditions—down-regulation (distance), maintenance (permit), and up-regulation (intensify)—for negative and neutral pictures, and investigated the neural responses during the stimulation and post-stimulation phase as well as during re-exposure after 10 min and after 1 week. We observed the following main results: first, we found greater activation in emotion-generating regions such as the amygdala in the permit vs. distance and the intensify vs. distance comparisons, but not in the intensify vs. permit comparison. Second, we observed greater activation in emotion-regulating regions such as the right inferior parietal and right superior / middle frontal cortex in the distance vs. permit and the distance vs. intensify contrasts, but not the permit vs. intensify contrast. Third, we found that the activation difference between distance and intensify within the amygdala reversed after the regulation period. Fourth, previous emotion regulation did not influence the activation during re-exposure, neither after 10 min nor after 1 week. Taken together, the results provide a partial replication of persistent effects observed in our preceding study, indicate different neural systems for up- and down-regulation, and demonstrate that a broader perspective on emotion regulation can be achieved by simultaneously considering different goals, directions, and strategies of emotion regulation in a single experiment.

## 1 Introduction

Cognitive emotion regulation refers to all processes through which individuals modulate their emotions consciously or unconsciously to appropriately respond to environmental demands (Gross, 1998; McRae & Gross, 2020). Down-regulation of negative emotions is often argued to be the most adaptive form of emotion regulation, whereas up-regulation of negative emotions—for example, by cognitive strategies such as worrying or rumination—is a key feature of several mental disorders (Aldao, Nolen-Hoeksema, & Schweizer, 2010). Adaptiveness, however, depends on context, and even the up-regulation of negative emotions can have beneficial effects, for example, when having to cope with a threat, or in the context of exposure therapy in the treatment of anxiety disorders. The notion that even negative emotions can be adaptive suggests a broader perspective on emotion regulation. This study proposes that this can be achieved by simultaneously considering different goals, directions, and strategies of emotion regulation in a single experiment.

The Process Model of Emotion Regulation and its subsequent taxonomy offer a comprehensive framework for understanding different strategies and tactics used in emotion regulation (Gross, 2014; Powers & LaBar, 2019). The model distinguishes five main strategies of emotion regulation based on when they are implemented in the emotion generation process: Situation selection, situation modification, attentional deployment, and cognitive change (reappraisal). In this study, the focus centers on cognitive change, specifically the technique known as reappraisal. Reappraisal has been consistently highlighted as an adaptive method for regulating emotions, as supported by numerous reports indicating its effectiveness in the short term, as well as its relevance to understanding psychopathological conditions (Aldao et al., 2010; Powers & LaBar, 2019; Webb, Miles, & Sheeran, 2012). Reappraisal operates at an early stage within the emotion generation process (antecedent phase). Its core principle involves modifying the emotional value of a stimulus that triggers the emotion. Moreover, reappraisal can be further dissected into distinct tactics (Powers & LaBar, 2019). These tactics include reinterpretation, encompassing the act of altering the interpretation or meaning of a stimulus, and distancing, previously often referred to as detachment. Notably, a meta-analysis conducted by Webb et al. (2012) revealed that while reappraisal overall demonstrates effectiveness, distancing (with an effect size of d+ = 0.45) exhibits greater advantages than reinterpretation (with an effect size of d+ = 0.36). Distancing can be achieved by adopting perspectives that are spatially distant from the stimulus, temporally distant, involve hypothetical scenarios rather than real events, or adopt an objective standpoint. Most studies, including our own, which have explored distancing using neuroimaging methodologies, have so far employed the objective form. In this design, participants are instructed to assume the perspective of an impartial and objective observer in order to regulate their emotional responses (Walter et al., 2009).

Up-regulation of emotions is often used as an umbrella term for different regulation strategies that serve the purpose of increasing or enhancing the subjective experience of a particular emotional state (Morawetz, Bode, Derntl, & Heekeren, 2017; Ochsner et al., 2004). One way to achieve this is the intensification of an emotional experience, for example, by assuming the position of a person directly involved in an emotionally intense situation. This form of intensification corresponds to a reappraisal strategy that focuses on re-interpreting the self-relevance of pictured events. Another way is the reinterpretation of an emotional event, for example, by exaggerating its unpleasant aspects into catastrophic consequences (Ochsner et al., 2004). There might be more and alternative ways to up-regulate emotions, since this concept is considerably less elaborated than down-regulation; consequently, a framework that incorporates changes of emotional state into both directions is lacking so far (Min et al., 2022; Morawetz et al., 2017). Emotional up- and down-regulation summarize a number of different regulation strategies that may not necessarily mirror each other (Ochsner et al., 2004): for example, reinterpretation can both increase and decrease the subjective experience of an emotion and its psychophysiological correlates (Ray, McRae, Ochsner, & Gross, 2010), but it is unclear whether or not expressive suppression or distraction of attention, two other strategies commonly implicated in the down-regulation of emotions (Gross, 1998), have up-regulation counterparts. One may imagine that processes such as the exaggeration of facial expressions or the focusing of attention may enhance the experience of an emotion, but since evidence is lacking, it is premature to assume that up- and down-regulation strategies are two ends of a common continuum. Some process characteristics such as attentional control, cognitive change, and behavorial modification, however, apply to both directions of regulation (Ochsner et al., 2004); thus, from a conceptual point of view, up-regulation shares both similarities and differences with down-regulation.

### 1.1 Neuroimaging studies on up- and down-regulation

It is a matter of ongoing research how these process characteristics are reflected on a neural level. Specifically, it is of interest (1) which brain regions are associated with up- and down-regulation, and (2) what distinguishes up- and down-regulation on a neural level (Frank et al., 2014; Morawetz et al., 2017). The first question (1) concerns a general regulation effect that is typically tested against a baseline condition which does not involve regulation. The second question (2) is a differential contrast, where the two conditions are compared with each other. One important implication of this distinction is that neither approach alone is sufficient to both describe and distinguish up- and down-regulation; instead, only a joint consideration of both contrasts can give a complete picture about the brain activation changes in response to the experimental conditions.

Early studies investigating the up- and down-regulation of negative emotions conclude that both variants involve prefrontal and anterior cingulate regions, that amygdala activation is modulated according to the goal of the regulation, and that some regions appear to be uniquely recruited by either form of regulation: the left rostromedial prefrontal cortex (PFC) in the case of up-regulation, and the right lateral and orbital PFC in the case of down-regulation (Ochsner et al., 2004). This general pattern has been confirmed by other studies, for example, Eippert et al. (2007) and Kim and Hamann (2007). Both found a bidirectional modulation of the amygdala according to the goal of the regulation. Both also found common and unique patterns of cortical activation during regulation, albeit with differences regarding the location and lateralization of these activations. Specifically, activation of the left anterior cingulate cortex, dorsolateral prefrontal cortex, and orbitofrontal cortex during down-regulation was found in one study (Eippert et al., 2007), but activation of the bilateral prefrontal cortex was found in the other (Kim & Hamann, 2007). For up-regulation, unique activation either of the bilateral prefrontal cortex or of left prefrontal regions has been reported. Furthermore, in a recent study, Min et al. (2022) observed that while up- and down-regulation both recruited regulatory regions in the frontal and cingulate cortex, they acted on distinct affect-generating regions in different ways: up-regulation was associated with increased activity in the amygdala, anterior insula, striatum, and anterior cingulate gyrus, whereas down-regulation was associated with decreased activity in the posterior insula and postcentral gyrus. This challenges the so-called *affective dial hypothesis*, that is, the idea that up- and down-regulation modulates the same affect-generating region in opposite directions (Min et al., 2022). Taken together, this illustrates that there is only partial overlap between the results of different empirical studies.

Discrepancies of this kind can be resolved by meta-analytic approaches, which have addressed which regions are involved in up-regulation in general, and also which activation patterns specifically distinguish up- and down-regulation. For comparisons of the first kind, that is, addressing general regulation effects, one meta-analysis identified the left superior frontal gyrus and left supplementary motor area, the bilateral insula, precentral gyrus, and midcingulate cortex, and the left thalamus and right globus pallidus (Frank et al., 2014). Another analysis confirmed the involvement of the supplementary motor area, but also implicated the bilateral ventrolateral and dorsomedial prefrontal cortex, and the putamen in emotional up-regulation (Morawetz et al., 2017). Remarkably, concurrent activation increases in the amygdala were evident only in Frank et al. (2014). Comparisons of the second kind, that is, differential regulation effects, help in identifying distinct activation patterns between up- and down-regulation. In this regard, Frank et al. (2014) found activations of the left amygdala and parahippocampal gyrus to differ between the two regulation conditions, with activation decreases for down-regulation and increases for up-regulation in both regions. Morawetz et al. (2017) found that the supplementary motor area and the insula were more often activated during the increasing of emotions, and that the right superior and middle frontal as well as right inferior parietal regions were more likely to be activated during the decreasing of emotions. A caveat in these analyses that impedes a systematic comparison is that up-regulation is often associated with positive emotions, whereas down-regulation typically focuses on negative emotions. To our knowledge, so far only a single, non-quantitative review has resolved these confounds, indicating activation of the left cortical hemisphere for reappraisal of both positive and negative stimuli, but activation of the right hemisphere primarily for reappraisal of negative stimuli (Ochsner, Silvers, & Buhle, 2012). Leaving these issues aside, the available meta-analytic evidence suggests the involvement of a core set of frontal and cingulate cortical regions in both up- and down-regulation. It also highlights the pivotal role of the amygdala in the generation of emotional responses and as a target of top-down modulation. Open questions remain about common vs. distinct patterns of cortical activation during regulation, and also about how top-down signals specifically impact amygdala activation—for example, with regard to intensity or duration of the emotional response.

### 1.2 Temporal effects of up- and down-regulation

Several studies have also addressed the temporal aspects during emotional up- and down-regulation. In this regard, most of the existing knowledge concerns down-regulation, for which persisting regulation effects across both short and long time-scales have been demonstrated (Walter et al., 2009). In contrast, little is known about up-regulation. Hermann, Kress, and Stark (2017), however, investigated the immediate and prolonged effects of both emotional up- and down-regulation. Lasting experiential and neural effects were only observed for down-regulation of negative feelings via reappraisal, but not for other strategies (distraction) or other goals of regulation (up-regulation). Further, the lasting neural effects were confined to prefrontal regions, and were not observed in the amygdala. Another study by Hermann and colleagues (2020) investigated temporal effects of down-regulation during passive re-exposure after 1 week. They found lasting effects for reinterpretation (i.e., stronger activation of amygdala and ventromedial prefrontal cortex, reduced negative feelings), but not for distancing. In contrast, in previous studies that included emotional down-regulation, but not up-regulation, we and others (Diers et al., 2021; Walter et al., 2009) observed a partial reversal of regulation effects immediately after the stimulation phase as well as during immediate and prolonged re-exposure experiments.

### 1.3 Aims and rationale of the present study

Taken together, the evidence to identify common or distinct spatial and temporal neural activation patterns in emotional up-regulation is still scarce. Further, a particular problem is that up-regulation is commonly associated with positive stimuli, and down-regulation with negative stimuli. This confounding precludes insights into the question if there is a general regulation capacity, or if emotional regulation is dependent on its goal and direction. Hence, our study targets the main question if neuroimaging findings associated with down-regulation generalize to up-regulation, or if there are different neural systems implicated in up- and down-regulation. A test of this question requires a full factorial design with exhaustive combinations of all stimulus classes and all regulation conditions. This allows to address the following more detailed aims: first, to determine if up- and down-regulation rely on same or different neural systems; and which activation patterns within these systems distinguish these two strategies. Second, to characterize the temporal effects of these activations; specifically, to determine whether or not there are any short- or long-term lasting effects, and if so, whether these differ between up- and down-regulation. Finally, to replicate and possibly differentiate or generalize our previous results in the same experimental paradigm without an up-regulation condition (Diers et al., 2021). To this end, we implement an experimental design that contrasts up- and down-regulation with each other and with a no-regulate (“permit”) control condition. We use neutral and negative stimuli during all conditions, and a slow event-related design with follow-up measurements after 10 min and after 1 week. Due to our previous results as well as the results of others, the amygdala is a particular region of interest in this study, in conjunction with the cortical brain areas previously implicated in emotional up- and down-regulation.

## 2 Materials and Methods

### 2.1 Study design

This study presents data collected within a larger project on neural correlates and individual differences in emotion regulation and its aftereffects (CRC 940 Project A5). A preceding study on regulatory and post-regulatory effects of emotion down-regulation has already been published (Diers et al., 2021). The study presented here used a similar design but extended the paradigm with an up-regulation condition in a separate sample, and acquired data in a non-overlapping sample of participants. Some of the data reported in this article have been re-used in three follow-up studies (see Fig. S1 for an overview; Figures and Tables starting with “S” can be found in the Supplementary Material): in accordance with the a priori specified analysis plan (http://gepris.dfg.de/gepris/projekt/223659428 and https://tu-dresden.de/bereichsuebergreifendes/sfb940/research/a-mechanismen/a5), associations with genetic polymorphisms were investigated (Gärtner et al., 2019) as well as the relation between emotion regulation and personality (Scheffel et al., 2019). Additionally, associations of emotion regulation success and dispositional emotion regulation with resting-state cortico-limbic connectivity have been analyzed (Dörfel, Gärtner, & Scheffel, 2020). Results from the present sample on the research questions of this publication have not been reported in any of these publications, and are independent from those in Diers et al. (2021). We report how we determined our sample size, all data exclusions (if any), all manipulations, and all measures in our study, as recommended by transparency guidelines (Simmons, Nelson, & Simonsohn, 2012). All procedures performed in studies involving human participants were in accordance with the ethical standards of the institutional and national research committee and with the 1964 Helsinki declaration and its later amendments or comparable ethical standards. The experimental protocol was approved by the ethics committee of the Technische Universität Dresden (EK10012012). Data and materials are provided at the Open Science Framework (https://osf.io/ktjnw/).

### 2.2 Participants

Forty-seven volunteers (22 male, age range 18−36 years, age
mean±SD=25.2±4.4 years) were recruited from the university community. All 47 participants completed the first session of the experiment. Out of these 47 participants, 30 returned within a period of 6−10 days for their second MRI measurement. Sample size was defined based on feasibility considerations. This resulted in a target sample size of approximately 48 participants. The sample size that we considered feasible to collect enabled us to at least detect medium-sized effects at a significance level of 0.05 and a power of 0.80—at least for the main experiment. In contrast, the attrition of participants for the follow-up experiment limits statistical power for the second part of the study, where we can only detect medium-to-large-sized effects. Despite high attrition, we found no demographic differences nor differences in the arousal ratings between participants who completed follow-up visits and those who discontinued the study (see Fig. S6 in the Supplementary Material). All participants were right-handed and did not report any current or prior neurological or psychiatric illness or treatment. All participants provided written informed consent for being included in the study and received financial compensation for their time and effort.

### 2.3 Experimental paradigm and procedure

The study consisted of two sessions, 1 week apart; for detailed descriptions, see Dörfel et al. (2020) and Scheffel et al. (2019). During the first session (70 min), participants performed a preparatory scan (5 min), four runs of an emotion regulation task (44 min), an anatomical scan (8 min), and a re-exposure task (12 min). During the second session (30-40 min), participants performed a preparatory scan (5 min), a resting-state measurement (8 min) and repeated the re-exposure task (12 min). Additionally, participants were asked to fill in questionnaires with regard to individual differences in emotion regulation and their subjective experience during the fMRI measurement as well as provided a blood sample, which are not focused on in the present publication; for a detailed description, see Gärtner et al. (2019) and Scheffel et al. (2019).

#### 2.3.1 Emotion regulation task (timepoints 1 and 2)

During the emotion regulation task, participants were asked to either up-regulate, not to regulate, or to down-regulate their emotions arising in response to a set of negative and neutral pictures, where each picture was associated with one of the following instructions: for up-regulation, participants were asked to intensify their current emotions and bodily sensations (“intensify” condition). This increase should be achieved by imagining a close personal or physical involvement with the situation, and becoming aware of one’s own reactions to it. For maintenance of their emotions, participants were asked to adhere to a “permit” instruction, that is, to take a close look at the picture and permit any emotions that might arise as a result. They were encouraged to imagine immediately witnessing the depicted situation. During the “distance” condition, they were asked to “take the position of a non-involved observer, thinking about the picture in a neutral way.” This could be achieved, for example, by reducing the personal involvement with the depicted situation, for example, by assuming a personal or physical distance. The “distance” and “permit” strategies are identical to those in our previous work (Diers et al., 2021; Gärtner, Jawinski, & Strobel, 2023). In all conditions, participants were asked to refrain from interpreting the situation as not real, attaching a different meaning to the situation, or distracting themselves. All participants received written instructions including examples, completed a training session outside the MR scanner, which took about 10-15 min and consisted of 24 trials, and were interviewed about how they implemented the proposed emotion regulation strategies.

**Fig. 1.**
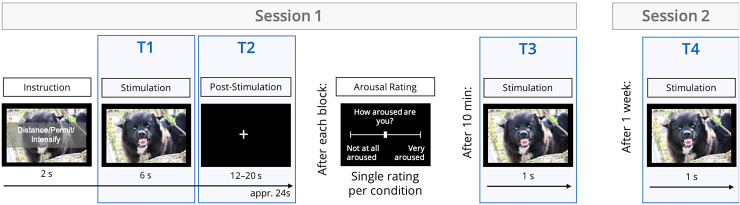
Schematic procedure of the emotion regulation task over all timepoints. The sample picture was taken from the Open Affective Standardized Image Set (OASIS; Kurdi et al., 2017) and was not used in the experiment.

Each of the four runs of the main emotion regulation experiment consisted of 24 trials, encompassing four trials for each condition. At the beginning of each trial, a picture was presented for 8000 ms. During the initial 2000 ms of this period, a semi-transparent overlay was presented across the center of the picture, which contained, as a single word, the instruction for either the “intensify,” the “permit,” or the “distance” condition. Following the offset of the picture, a fixation cross was presented for a variable period of 12-20 s. This rather long period was inserted into the trial to provide the participants with a relaxation phase, and to allow the return of the BOLD response to baseline levels. Altogether, the total duration of a single trial was, on average, 24 s. At the end of each run, participants were asked to give a rating of their retrospective subjective arousal. For each experimental condition, participants rated on a continuous scale, ranging from “not at all aroused” to “very highly aroused,” how much aroused they felt during the presentation of the negative and neutral pictures and the “distance,” “intensify,” and “permit” instructions, respectively. A schematic procedure of the task can be seen in Figure 1.

#### 2.3.2 Re-exposure task (timepoints 3 and 4)

The re-exposure task at the end of session 1 (timepoint 3, 10 min later) and during session 2 (timepoint 4, 1 week later) consisted of the presentation of exactly those negative and neutral pictures that participants had seen during the emotion regulation task. However, the order of picture presentation was different, the duration was shortened to 1000 ms, followed by a gap of 2.5 s to 27.5 s, and participants had no particular task except passively viewing the pictures. Specifically, they were instructed not to voluntarily change their emotional experience as they had done during the emotion regulation task. A schematic procedure of the task can be seen in Figure 1.

#### 2.3.3 Stimuli

Stimuli were selected from the International Affective Picture System (Lang, Bradley, & Cuthbert, 1997) and the EmoPics picture set (Wessa et al., 2010). We used three sets of negative pictures and three sets of neutral pictures (16 pictures per set), which were matched for content, arousal, and valence, respectively (mean valence of negative pictures: set1=2.71, set2=2.65, set
3=2.65; mean arousal of negative pictures: set 1 = 5.85, set2=5.69, set3=5.55; mean valence for neutral pictures: set1=5.17, set2=5.13, set3=5.19; mean arousal for neutral pictures: set1=2.94, set2=2.96, set3=2.85). We refrained from using positive pictures for economical reasons, to avoid excessive demand on the participants and effects of exhaustion. The negative pictures consisted primarily of depictions of animals, bodies, disaster, disgust, injuries, suffering, or violence, while the neutral pictures depicted various scenes, objects, and people. The negative and neutral picture sets were matched with regard to depictions of faces, other parts of the body, single or multiple persons, animals, and inanimate objects. In order to rule out any further stimulus- or content-related confounds, the sets of negative and neutral pictures as well as their assignment to the “distance,” “permit,” or “intensify” conditions were counterbalanced across participants. All pictures were presented onto a back-projection screen located at the rear end of the scanner and were viewed through a mirror attached to the head coil.

### 2.4 Data acquisition

Magnetic resonance (MR) imaging was done on a 3 Tesla scanner (Siemens Trio; Siemens Erlangen, Germany), using a 12-channel head coil. Functional (T2*) MR images were acquired using an EPI sequence with 42 axial slices (slice thickness 2 mm) per volume (TR 2410 ms; TE 25 ms; flip angle 80°; slice gap 1 mm; field of view 192  mm×192  mm; matrix size 64×64). In addition, anatomical (T1) images were acquired using an MPRAGE sequence that consisted of 176 sagittal slices with a thickness of 1 mm (TR 1900 ms; TE 2.26 ms; flip angle 9°; FOV 256  mm×256  mm; matrix size 256×256).

### 2.5 Data analysis

#### 2.5.1 Behavioral data analysis

Behavioral data analyses, that is, evaluation of the subjective ratings and possible relations between subjective and physiological measures, were performed using R 3.0.2 (http://r-project.org) including the ggplot2 package (Wickham, 2009), and generally consisted of a two-way repeated-measures ANOVA with subsequent post-hoc t-tests for dependent samples. All analyses, except those within SPM8, were conducted as two-tailed tests.

#### 2.5.2 fMRI analyses

Imaging data analysis was performed using Matlab 7.4 (MathWorks, Natick, MA) and SPM 8 (http://www.fil.ion.ucl.ac.uk/spm/software/spm8). After discarding the first four volumes of each run, preprocessing consisted of motion correction, coregistration of individual functional and anatomical data, spatial normalization of the anatomical images to the MNI template, application of the estimated transformation parameters to the coregistered functional images using a resampling resolution of $2\times 2\times 2{\text{ mm}}^{3}$, and spatial smoothing of the functional images (FWHM 8 mm).

First-level statistical analysis of the emotion regulation task was performed using a general linear model with regressors based on the experimental conditions, as detailed below, as well as six additional motion regressors of no interest. A standard high-pass filter of 0.0078 Hz (128 s) was also applied. To account for the notion that “rest” is not necessarily a true rest, especially not in emotionally challenging experimental paradigms (Lamke et al., 2014), we included not only the stimulation phase, but also the post-stimulation phase in our model, starting with the offset of each picture, and lasting for the same duration as the stimulation phase (either 0 s or 8 s). This resulted in two first-level models with 12 regressors of interest: we modeled the “permit neutral,” “permit negative,” “distance neutral,” “distance negative,” “intensify neutral,” and “intensify negative” conditions for both the stimulation (onset of picture presentation, timepoint 1) and post-stimulation (offset of picture presentation, timepoint 2) phase. As the temporal dynamics of amygdala activation may differ from those in cortical regions, we conducted an additional sensitivity analysis for the amygdala, where activation was modeled by a stick function (transient response) in addition to a boxcar function (sustained response). This resulted in two different first-level models for the amygdala, whereas a single first-level model was used for all other brain regions. In both cases, all regressors of interest were convolved with the canonical HRF, and the default high-pass filter for SPM8 (128 s) was used. The four imaging runs of the emotion regulation task were combined within one fixed-effects model. Resulting parameter estimates of interest were averaged across runs, submitted to a second-level, random-effects analysis, and evaluated using F-tests and t-tests for repeated measures. At the second level, we used two different statistical models: first, a two-way ANOVA with the factors “picture” (two levels; negative and neutral) and “regulation” (three levels: distance, permit, intensify) in order to assess the effects during the stimulation phase, which we refer to as “Model 1” in the results section. Second, another two-way ANOVA with the factors “regulation” (three levels: distance, permit, intensify) and “time” (two levels: stimulation, post-stimulation), which was restricted to negative stimuli and was conducted in order to elucidate temporal effects, that is, differences between the stimulation and post-stimulation phase. We refer to this model as “Model 2.”

The first-level model of the re-exposure runs (timepoints 3 and 4) included six regressors, which distinguished between neutral and negative stimuli and, additionally, those stimuli that had been presented with a “distance” instruction, a “permit” instruction, or an “intensify” instruction during the emotion regulation task. The duration of all events was set to 1000 ms.

Based on our a priori hypotheses, we employed two regions of interest, the left and right amygdala as defined by the Harvard-Oxford Subcortical Structural Atlas within the FSL software package (https://fsl.fmrib.ox.ac.uk/fsl/fslwiki/Atlases). For these analyses, we applied a voxel-wise threshold of p=.05 FWE after correction for small volume, and only activations with k>5 voxels are reported. For all other analyses, a voxel-wise threshold of p=.05 FWE with activation clusters k>25 voxels across the whole brain was applied. Activations were labeled using the Harvard-Oxford Structural Atlases as well as the Anatomy Toolbox for SPM8 (Eickhoff et al., 2005).

Since voxel-wise analyses are limited in integrating data across a predefined anatomical structure, we additionally obtained summary measures of activation within the left and right amygdala. For this purpose, we extracted parameter estimates for all experimental conditions from the individual first-level analyses using SPM8’s spm_summarise() function, and further analyzed these data using repeated-measures ANOVAs with the factors “picture,” “regulation,” and / or “time.” In addition, we extracted the activation time courses from these regions by using the rfxplot toolbox (http://rfxplot.sourceforge.net), which served as a descriptive illustration of the results of the model-based analyses (Gläscher, 2009).

## 3 Results

The results section is structured as follows: we first report the behavioral results, that is, the effects of the “picture” and “regulation” factors on the subjective arousal ratings, which were observed during timepoint 1 (i.e., during the stimulation phase of the emotion regulation task). This serves as a manipulation check to establish the validity of the experiment.

This is followed by a description of the effects of the “picture” and “regulation” factors on BOLD activation levels during timepoint 1. This analysis targets our first main research question, that is, to determine if up- and down-regulation rely on same or different neural systems; and which activation patterns within these systems distinguish these two strategies.

We then describe the effects of the factors “regulation” and “time” on BOLD activation levels during the stimulation phase (timepoint 1) and post-stimulation phase (timepoint 2). This analysis is motivated by our second main research question, that is, to characterize the temporal effects of emotion regulation, and focuses on the main and interaction effects in the emotion regulation task.

We finally report the impact of previous emotion regulation on BOLD activation during the re-exposure tasks after 10 min (timepoint 3) and after 1 week (timepoint 4) in order to assess any potential short- and long-term effects of previous emotion regulation. This analysis is also motivated by the research question regarding the temporal effects, but focuses on the re-exposure tasks instead of the emotion regulation task.

### 3.1 Behavioral analysis of the stimulation phase (timepoint 1)

The retrospective arousal ratings after each run (Figs. S2 and S3) demonstrated an interaction between picture and regulation (F(2,92)=4.09, p=.020, $\eta^{2}=.001$) as well as the main effects of picture (F(1,46)=126.01, p<.001, $\eta^{2}=.344$) and regulation (F(2,92)=70.53, p<.001, $\eta^{2}=.074$). Pairwise comparisons revealed that negative pictures were associated with higher subjective arousal ratings than neutral pictures; that pictures during intensify were rated as more arousing than pictures during permit; that pictures during permit were more arousing than pictures during distancing; and that differences between intensify, permit, and distance were slightly more pronounced for negative than neutral pictures (Table S5).

### 3.2 Activation differences during the stimulation phase (timepoint 1)

In this section, we report the main and interaction effects of the “picture” (negative vs. neutral) and “regulation” (distance vs. permit vs. intensify) factors during the stimulation phase, and subsequent pairwise comparisons between the different regulation conditions. This analysis is based on Model 1.

### 3.2.1 Activation differences between negative and neutral pictures

During the stimulation phase, the main effects of “picture” and “regulation” were observed in multiple brain regions (Table S1). In particular, the main effects of “picture” were present in extended regions of the occipital cortex, left and right temporal cortex, the left inferior parietal cortex, and midline structures. The left and right amygdala ROI demonstrated picture effects for both transient and sustained responses.

### 3.2.2 Activation differences between the three regulation strategies

Similar to the main effects of “picture,” main effects of “regulation” were distributed, with activation clusters in the right inferior parietal lobe, the right superior and middle frontal gyrus, and the occipital cortex, among others (Table S1). Within the left and right amygdala ROI, regulation effects were present for both transient and sustained responses. Similar effects were also observed when the analysis was restricted to negative pictures (Table S2A) or, although less pronounced, to neutral pictures (Table S2B).

### 3.2.3 Pairwise comparisons betwen regulation strategies

**Fig. 2.**
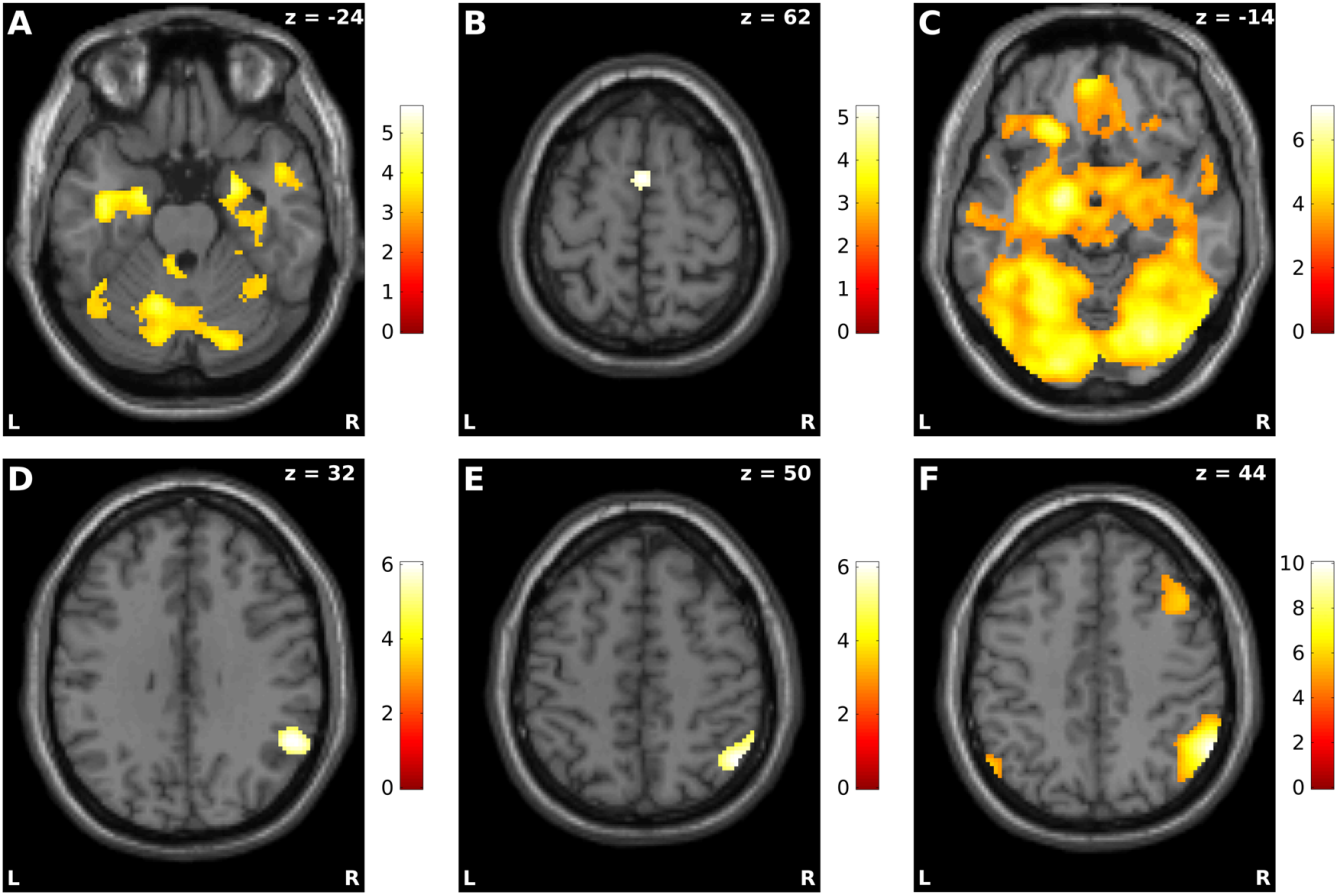
Whole-brain analysis of regulation effects. All analyses are restricted to negative stimuli. Shown are those sections that contain the activation maxima of the following contrasts: (A) permit > distance contrast for transient responses, (B) distance > permit contrast for sustained responses, (C) intensify > permit contrast for sustained responses, (D) permit > intensify contrast for sustained responses, (E) intensify > distance contrast for transient responses, and (F) distance > intensify contrast for sustained responses. The color scales represent t-statistics of the corresponding contrast.

Pairwise comparisons between regulation strategies. We next conducted directed comparisons between pairs of the different regulation conditions (Table 1): first, a comparison between the distance and permit conditions revealed greater activation during the permit condition in the occipital cortex as well as in the left and right amygdala ROI (both sustained and transient responses). The reverse effects, that is, greater activation for the distance condition, were present in the right angular gyrus and the right superior frontal gyrus. Similar patterns of results were observed when the analyses were restricted to either negative (Fig. 2A and 2B; Table S2A) or neutral pictures (Table S2B). Second, a comparison of the intensify and permit conditions showed greater activation during intensification in left precentral, frontal, and occipital areas, as well as right cerebellum, but not in the left or right amygdala ROI, neither for sustained nor for transient responses, nor for separate analyses of negative (Fig. 2C; Table S2A) or neutral pictures (Table S2B). The opposite contrast, that is, greater activation for the permit condition, identified the right angular gyrus, but no other brain region. This effect was also present in a separate analysis of negative pictures (Fig. 2D; Table S2A), but did not appear for neutral pictures (Table S2B). Third, activation increases in the distance condition as compared to the intensify condition were observed in the right inferior parietal cortex, right middle frontal gyrus, left angular gyrus, and the precuneus. Conversely, greater activation for the intensify condition was found in the left and right amygdala ROI (both sustained and transient) as well as the right occipital cortex. The general pattern of these results was also present in separate analyses of negative (Fig. 2E and 2F; Table S2A), but notably also neutral pictures (Table S2B).

### 3.2.4 Common effects of regulation conditions

Finally, we also evaluated possible common regulation effects, that is, a combination of the two regulation conditions “distance” and “intensify” vs. the non-regulating permit condition (Table 1). The regulation conditions led to greater activation in the left supplementary motor area and precentral gyrus, and the reverse contrast led to activation increases in the bilateral occipital gyrus and the right inferior temporal gyrus. No such effects were observed during separate analyses of either negative or neutral pictures, the only exception being occipital activation during the permit condition as compared to both regulation conditions when only neutral pictures were considered (Table S2A and S2B). The results reported so far can all be regarded as main effects of either “picture” or “regulation”. It is noteworthy that there were no interaction effects between “picture” and “regulation” in this analysis, neither as a global effect nor for any of the directed comparisons.

**Table 1.** Activation maxima during the emotion regulation task.

k	*p_FWE_*	*t*	*p_unc._*	x	y	z	Label
Distance *>* Permit							
1136	*<*0.001	8.75	*<*0.001	58	-50	32	Right Angular Gyrus
168	<0.001	6.13	*<*0.001	18	10	62	Right Superior Frontal Gyrus
89	<0.001	5.83	*<*0.001	-4	-24	26	Left Posterior Cingulate Cortex
65	0.002	5.39	*<*0.001	-60	-56	32	Left Supramarginal Gyrus
Permit *>* Distance							
1877	*<*0.001	9.74	*<*0.001	28	-94	0	Right Middle Occipital Gyrus
2074	*<*0.001	8.14	*<*0.001	-30	-90	-6	Left Middle Occipital Gyrus
136	*<*0.001	4.25	*<*0.001	-20	-6	-18	Left Amygdala (ROI, sustained)
130	0.003	3.76	*<*0.001	20	-4	-18	Right Amygdala (ROI, sustained)
76	0.003	3.76	*<*0.001	-24	-4	-26	Left Amygdala (ROI, transient)
40	0.002	3.96	*<*0.001	26	-2	-26	Right Amygdala (ROI, transient)
Permit *>* Intensify							
368	*<*0.001	6.06	*<*0.001	44	-60	54	Right Angular Gyrus
Intensify *>* Permit							
466	*<*0.001	6.38	*<*0.001	-8	10	50	Left Supplementary Motor Area
129	*<*0.001	5.84	*<*0.001	36	-64	-26	Right Cerebellum
95	0.004	5.22	*<*0.001	-38	-2	60	Left Precentral Gyrus
44	0.008	5.06	*<*0.001	-40	10	24	Left Inferior Frontal Gyrus
33	0.011	4.98	*<*0.001	-42	-74	26	Left Middle Occipital Gyrus
Intensify *>* Distance							
4147	*<*0.001	8.41	*<*0.001	30	-94	0	Right Middle Occipital Gyrus
25	0.010	5.01	*<*0.001	-14	-12	-16	N/A
84	*<*0.001	4.77	*<*0.001	-16	-10	-16	Left Amygdala (ROI, sustained)
46	0.006	3.56	*<*0.001	14	-8	-16	Right Amygdala (ROI, sustained)
210	*<*0.001	5.91	*<*0.001	-18	-8	-14	Left Amygdala (ROI, transient)
152	0.002	3.84	*<*0.001	24	-4	-18	Right Amygdala (ROI, transient)
Distance *>* Intensify							
1878	*<*0.001	10.96	*<*0.001	58	-54	40	Right Inferior Parietal Lobule
899	*<*0.001	6.70	*<*0.001	44	20	42	Right Middle Frontal Gyrus
111	*<*0.001	6.00	*<*0.001	-54	-62	40	Left Angular Gyrus
139	*<*0.001	5.96	*<*0.001	10	-66	36	Right Precuneus
170	*<*0.001	5.88	*<*0.001	4	-26	26	Right Posterior Cingulate Cortex
42	0.002	5.36	*<*0.001	48	42	-10	Right Inferior Frontal Gyrus
(Distance + Intensify) *>* Permit							
193	0.001	5.45	*<*0.001	-8	12	48	Left Supplementary Motor Area
86	0.004	5.20	*<*0.001	-36	0	54	Left Precentral Gyrus
Permit *>* (Distance + Intensify)							
208	*<*0.001	6.10	*<*0.001	28	-94	0	Right Middle Occipital Gyrus
84	0.002	5.38	*<*0.001	-28	-90	-4	Left Middle Occipital Gyrus
61	0.003	5.29	*<*0.001	46	-62	-6	Right Inferior Temporal Gyrus
(DistanceNeutral *>* PermitNeutral) *>* (DistanceNegative *>* PermitNegative)							
— *No results* —							
(PermitNeutral *>* DistanceNeutral) *>* (PermitNegative *>* DistanceNegative)							
— *No results* —							
(PermitNeutral *>* IntensifyNeutral) *>* (PermitNegative *>* IntensifyNegative)							
— *No results* —							
(IntensifyNeutral *>* PermitNeutral) *>* (IntensifyNegative *>* PermitNegative)							
— *No results* —							
(IntensifyNeutral *>* DistanceNeutral) *>* (IntensifyNegative *>* DistanceNegative)							
— *No results* —							
(DistanceNeutral *>* IntensifyNeutral) *>* (DistanceNegative *>* IntensifyNegative)							
— *No results* —							

Reported are results during the stimulation phase.

Abbreviations: k = spatial extent, *p_FWE_* = *p*-values corrected for multiple comparisons (FWE), *p_unc._* = uncorrected *p*-values, *t* = *t*-statistics, x, y, z = MNI coordinates. ROI indicates that an activation peak was observed within the left or right amygdala region of interest.

### 3.2.5 Analysis of summary statistics in the left and right amygdala ROIs

**Fig. 3.**
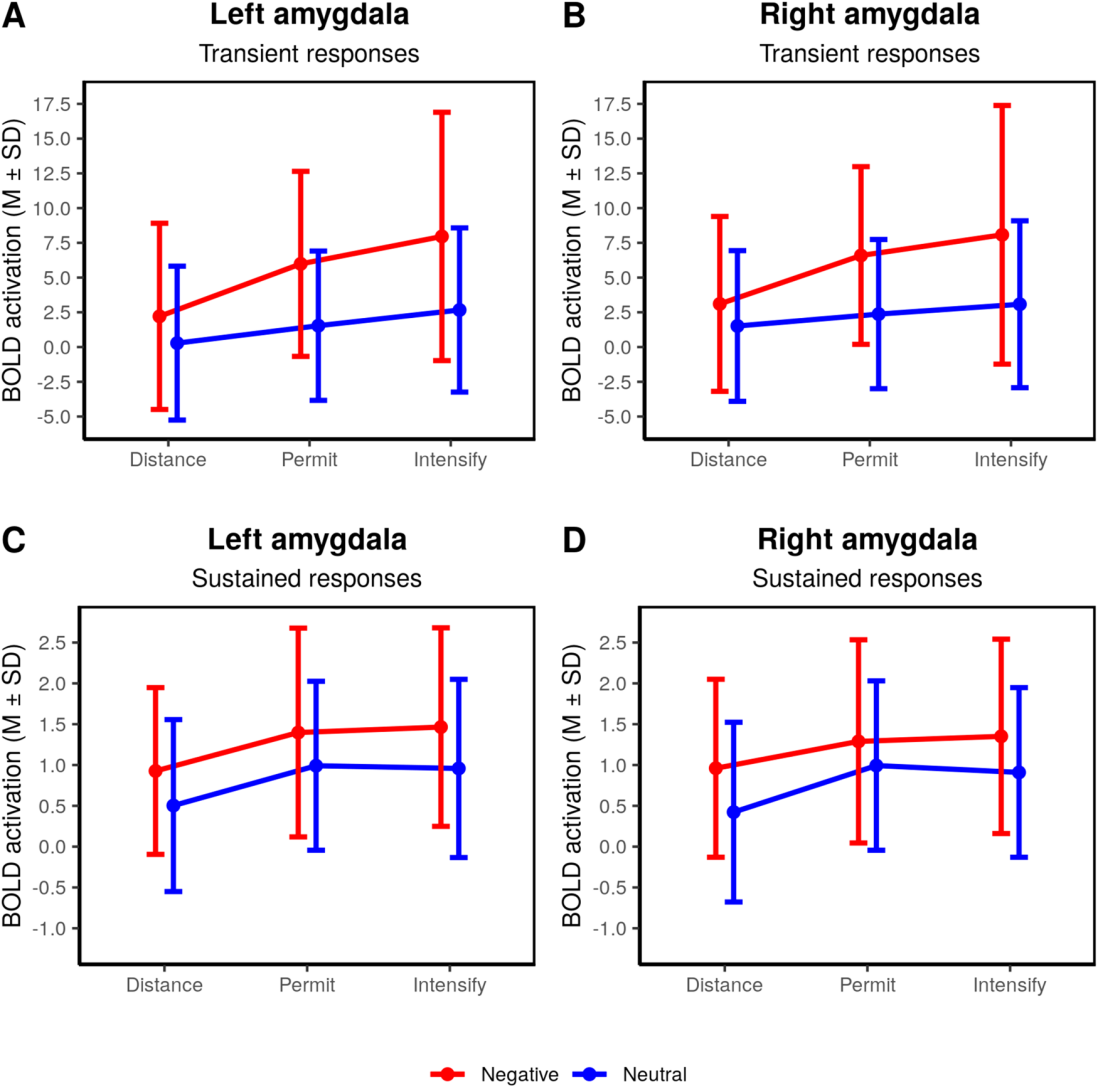
Summary statistics for the activation of the amygdala ROI during the stimulation phase. Lines represent mean and standard deviations for each condition.

We conducted an additional analysis of summary statistics based on the left and right amygdala ROIs in order to further characterize the effects observed during the voxel-based analysis. This analysis was done separately for transient and sustained responses in the left and right amygdala. For transient responses, we observed “picture” and “regulation” main effects as well as interaction effects during the stimulation phase (left amygdala: Picture: F(1,46)=34.28, p<.001, $\eta^{2}=.081$; Regulation: F(2,92)=10.40, p<.001, $\eta^{2}=.061$; Picture × Regulation: F(2,92)=3.24, p=.044, $\eta^{2}=.012$); right amygdala: Picture: F(1,46)=37.80, p<.001, $\eta^{2}=.071$; Regulation: F(2,92)=6.56, p=.002, $\eta^{2}=.041$; Picture × Regulation: F(2,92)=3.35, p=.039, $\eta^{2}=.012$; see Table S6 for pairwise comparisons). Figure 3A and 3B shows greater activation for negative than for neutral pictures, an almost linear increase in activation from distance to intensify in both the left and right amygdala, and a steeper slope of this increase for negative pictures. A slightly different pattern emerged for sustained responses. Here, we observed highly significant “picture” and “regulation” effects, but no interaction effects during the stimulation phase (left amygdala: Picture F(1,46)=15.13, p<.001, $\eta^{2}=.039$; Regulation: F(2,92)=9.24, p<.001, $\eta^{2}=.041$; Picture × Regulation: F(2,92)=0.12, p=.890, $\eta^{2}<.001$; right amygdala: Picture: F(1,46)=17.13, p<.001, $\eta^{2}=.036$; Regulation: F(2,92)=6.92, p=.002, $\eta^{2}=.034$; Picture × Regulation: F(2,92)=0.53, p=.588, $\eta^{2}=.002$; see Table S6 for pairwise comparisons). Figure 3C and 3D shows greater activation for negative than for neutral pictures as well as an increase in activation from distance to permit, but apparently not for permit to intensify.

### 3.3 Neuronal activation differences during the stimulation phase and post-stimulation phase (timepoints 1 and 2)

We next report differences between the regulation conditions during the stimulation and post-stimulation phases as well as the interaction effects between regulation and time, followed by pairwise comparisons between different regulation conditions. This analyis is based on Model 2, which contains the “regulation” and “time” factors, and restricts the analysis to negative stimuli.

#### 3.3.1 Main and interaction effects of the “time” and “regulation” factors

We observed main effects of “time” in a number of brain regions, including several frontal, occipital, parietal, and subcortical regions as well as the left and right amygdala ROI (Table S3). Main effects of “regulation” were also present in this analysis, but mainly in frontal and parietal regions as well as the precuneus, but not in the left or right amygdala. An overall interaction effect of “time” and “regulation” was observed in the amygdala ROI as well as the right inferior parietal cortex, the left supplementary motor area, and the left and right occipital cortex.

#### 3.3.2 Pairwise comparisons between regulation conditions

When looking at contrasts for specific regulation conditions (Table 2), we found a regulation-by-time interaction for the intensify and distance conditions in extended temporal and occipital regions as well as the left amygdala ROI (primarily transient activation). In these regions, the intensify minus distance contrast was greater during the stimulation phase than during the post-stimulation phase. The reverse effect, that is, greater activation during the post-stimulation than stimulation phase, was observed in the right inferior parietal lobe. Apart from that, for no other pair of regulation conditions the interaction effect was significant.

**Table 2:** Activation maxima during the emotion regulation task.

STIMULATION AND POST-STIMULATION PHASE (NEGATIVE STIMULI)							
K	*p_FWE_*	*t*	*p_unc._*	X	y	z	Label
(DistanceStimPhase *>* PermitStimPhase) *>* (DistancePostStimPhase *>* PermitPostStimPhase)							
— *No results* —							
(PermitStimPhase *>* DistanceStimPhase) *>* (PermitPostStimPhase *>* DistancePostStimPhase)							
5	0.010	3.31	0.001	-22	-4	-24	Left Amygdala (ROI, transient)
30	0.001	4.14	*<*0.001	26	-2	-26	Right Amygdala (ROI, transient)
(PermitStimPhase *>* IntensifyStimPhase) *>* (PermitPostStimPhase *>* IntensifyPostStimPhase)							
— *No results* —							
(IntensifyStimPhase *>* PermitStimPhase) *>* (IntensifyPostStimPhase *>* PermitPostStimPhase)							
— *No results* —							
(IntensifyStimPhase *>* DistanceStimPhase) *>* (IntensifyPostStimPhase *>* DistancePostStimPhase)							
217	*<*0.001	5.70	*<*0.001	-32	-90	-8	Left Inferior Occipital Gyrus
256	0.002	5.36	*<*0.001	30	-90	0	Right Middle Occipital Gyrus
69	0.003	5.25	*<*0.001	50	-70	-6	Right Inferior Temporal Gyrus
161	0.003	5.23	*<*0.001	-42	-68	-2	Left Middle Occipital Gyrus
45	0.003	5.20	*<*0.001	26	-62	14	Right Calcarine Gyrus
89	0.004	5.19	*<*0.001	-8	-94	-12	Left Calcarine Gyrus
188	0.004	5.16	*<*0.001	28	-72	34	Right Superior Occipital Gyrus
50	0.011	4.94	*<*0.001	0	-2	68	Left Supplementary Motor Area
51	0.013	4.89	*<*0.001	-26	-90	18	Left Middle Occipital Gyrus
38	0.013	4.88	*<*0.001	12	-82	36	Right Cuneus
42	*<*0.001	4.28	*<*0.001	-16	-10	-14	Left Amygdala (ROI, sustained)
206	*<*0.001	5.91	*<*0.001	-16	-4	-16	Left Amygdala (ROI, transient)
226	*<*0.001	4.31	*<*0.001	28	-4	-20	Right Amygdala (ROI, transient)
(DistanceStimPhase *>* IntensifyStimPhase) *>* (DistancePostStimPhase *>* IntensifyPostStimPhase)							
72	*<*0.001	6.45	*<*0.001	60	-52	42	Right Inferior Parietal Lobule

STIMULATION PHASE (NEGATIVE STIMULI)							
DistanceStimPhase *>* PermitStimPhase							
81	0.003	5.23	*<*0.001	54	-52	30	Right Angular Gyrus
PermitStimPhase *>* DistanceStimPhase							
115	*<*0.001	5.70	*<*0.001	32	-96	-2	Right Inferior Occipital Gyrus
105	0.001	5.47	*<*0.001	-28	-94	-6	Left Inferior Occipital Gyrus
112	*<*0.001	4.37	*<*0.001	-22	-4	-24	Left Amygdala (ROI, transient)
52	*<*0.001	4.35	*<*0.001	26	-2	-26	Right Amygdala (ROI, transient)
PermitStimPhase *>* IntensifyStimPhase							
351	*<*0.001	6.15	*<*0.001	56	-58	44	Right Inferior Parietal Lobule
IntensifyStimPhase *>* PermitStimPhase							
— *No results* —							
IntensifyStimPhase *>* DistanceStimPhase							
228	*<*0.001	5.79	*<*0.001	-28	-96	-8	Left Inferior Occipital Gyrus
169	*<*0.001	5.76	*<*0.001	30	-94	-2	Right Inferior Occipital Gyrus
13	0.002	3.75	*<*0.001	-16	-10	-14	Left Amygdala (ROI, sustained)
216	*<*0.001	5.92	*<*0.001	-16	-6	-16	Left Amygdala (ROI, transient)
248	*<*0.001	4.57	*<*0.001	18	-10	-12	Right Amygdala (ROI, transient)
DistanceStimPhase *>* IntensifyStimPhase							
1179	*<*0.001	9.43	*<*0.001	58	-56	42	Right Inferior Parietal Lobule
118	*<*0.001	5.98	*<*0.001	-54	-62	42	Left Angular Gyrus
126	0.002	5.35	*<*0.001	40	18	46	Right Middle Frontal Gyrus
72	0.005	5.10	*<*0.001	4	-26	26	Right Posterior Cingulate Cortex
50	0.009	4.98	*<*0.001	18	30	52	Right Superior Frontal Gyrus
57	0.009	4.96	*<*0.001	44	46	-12	Right Inferior Frontal Gyrus

POST-STIMULATION PHASE (NEGATIVE STIMULI)							
DistancePostStimPhase *>* PermitPostStimPhase							
— *No results* —							
PermitPostStimPhase *>* DistancePostStimPhase							
— *No results* —							
PermitPostStimPhase *>* IntensifyPostStimPhase							
— *No results* —							
IntensifyPostStimPhase *>* PermitPostStimPhase							
— *No results* —							
IntensifyPostStimPhase *>* DistancePostStimPhase							
— *No results* —							
DistancePostStimPhase *>* IntensifyPostStimPhase							
321	0.001	5.40	*<*0.001	-6	-78	30	Left Cuneus
34	0.011	4.92	*<*0.001	-2	-42	18	Left Posterior Cingulate Cortex
27	0.025	4.72	*<*0.001	26	-74	40	Right Superior Occipital Gyrus

Reported are interaction effects between the stimulation and post-stimulation phase as well as effects during either stimulation or post-stimulation phase. Abbreviations as in Table 1.

#### 3.3.3 Analyses restricted to either the stimulation or post-stimulation phase

We also restricted the analysis to either the stimulation or post-stimulation phase (Table 2). During the stimulation phase, we found greater amygdala (primarily transient) and occipital activation for both the permit and the intensify conditions as compared to the distance condition. Conversely, greater activation for the distance—but not the intensify condition—as compared to the permit condition was found in the right parietal and frontal cortex. During the post-stimulation phase, differences between regulation conditions were only observed for the distance and intensify conditions, with greater activation in the left cuneus and posterior cingulate cortex during distance than during intensify.

#### 3.3.4 Analysis of summary statistics in the left and right amygdala ROIs

**Fig. 4.**
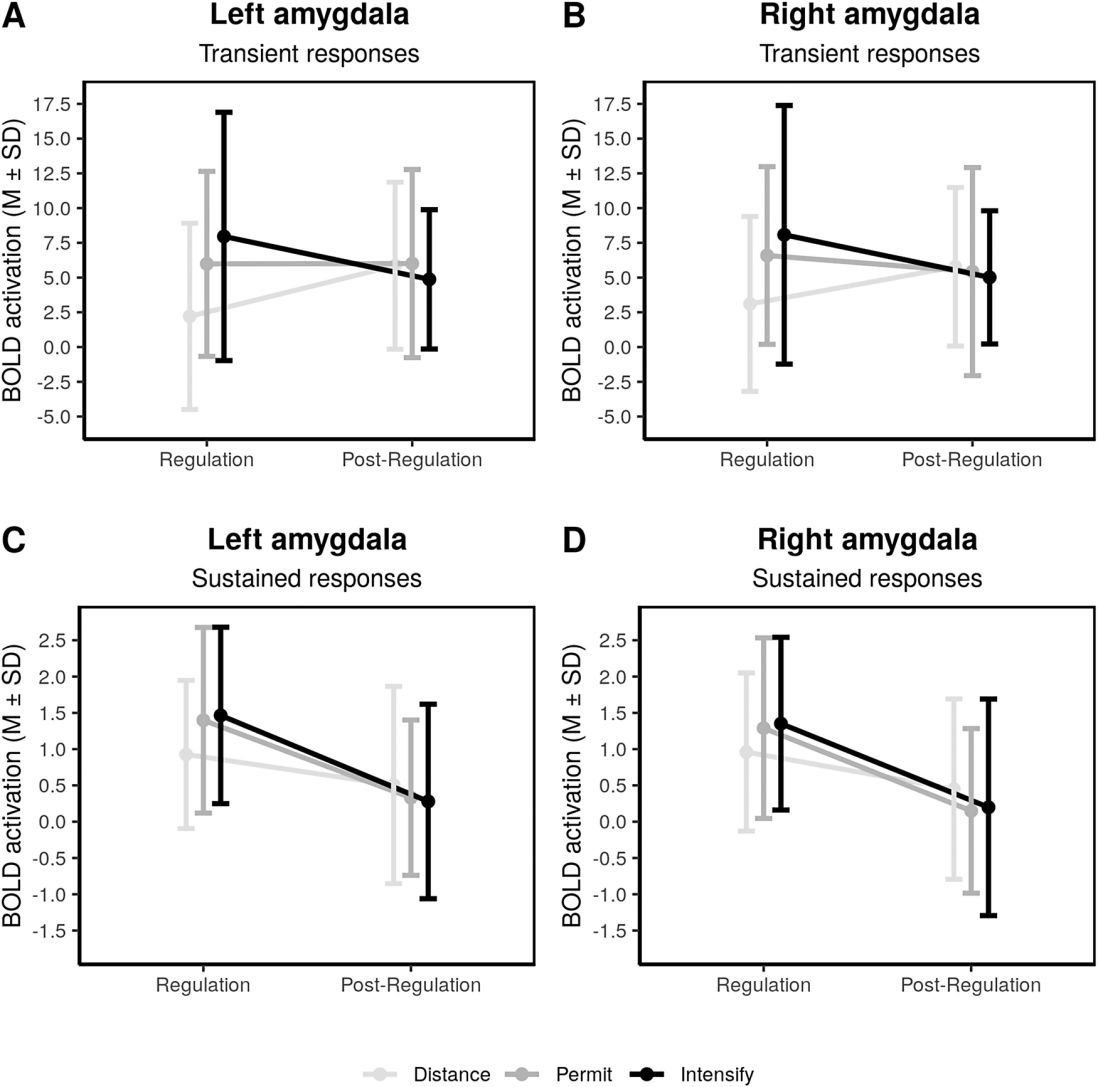
Summary statistics for the amygdala ROI during the stimulation phase (T1) and the post-stimulation phase (T2), for different regulation instructions in the stimulation phase (T1). This analysis is limited to negative stimuli. Lines represent mean and standard deviations for each condition.

For the left and right amygdala, we additionally conducted an analysis of summary statistics that was restricted to negative stimuli, but also took the effects of time—that is, the stimulation phase and the post-stimulation phase—into account. For transient responses, we observed significant regulation-by-time interactions as well as significant main effects of “regulation,” but not of “time” (left amygdala: Regulation: F(2,92)=5.42, p=.006, $\eta^{2}=.023$; Time: F(1,46)=0.05, p=.829, $\eta^{2}<.001$; Regulation × Time: F(2,92)=7.73, p=.001, $\eta^{2}=.040$; right amygdala: Regulation F(2,92)=3.31, p=.041, $\eta^{2}=.017$; Time: F(1,46)=0.29, p=.590, $\eta^{2}<.001$; Regulation × Time: F(2,92)=6.06, p=.003, $\eta^{2}=.030$; see Table S7 for pairwise comparisons). That is, when considering both the stimulation phase and the post-stimulation phase within one single analysis, we found that activation within the left and right amygdala was dependent on both the regulation strategy and the time of measurement: during the distance condition, we observed an increase of activation after the stimulation phase, while during the permit and intensify conditions, either constant or decreasing levels of activation were present (Fig. 4A and 4B). For sustained responses, there were significant main effects of “time,” and not “regulation,” but also regulation × time interaction effects (left amygdala: Regulation F(2,92)=0.67, p=.514, $\eta^{2}=.004$; Time: F(1,46)=32.88, p<.001, $\eta^{2}=.120$; Regulation × Time: F(2,92)=3.82, p=.026, $\eta^{2}=.019$; right amygdala: Regulation: F(2,92)=0.10, p=.902, $\eta^{2}=.001$; Time: F(1,46)=44.83, p<.001, $\eta^{2}=.127$; Regulation × Time F(2,92)=3.55, p=.033, $\eta^{2}=.015$; see Table S7 for pairwise comparisons). Altogether, this indicates a general decrease of activation over time, which was less pronounced after the distance condition (Fig. 4C and 4D).

#### 3.3.5 Activation time courses during the stimulation- and post-stimulation phase

**Fig. 5.**
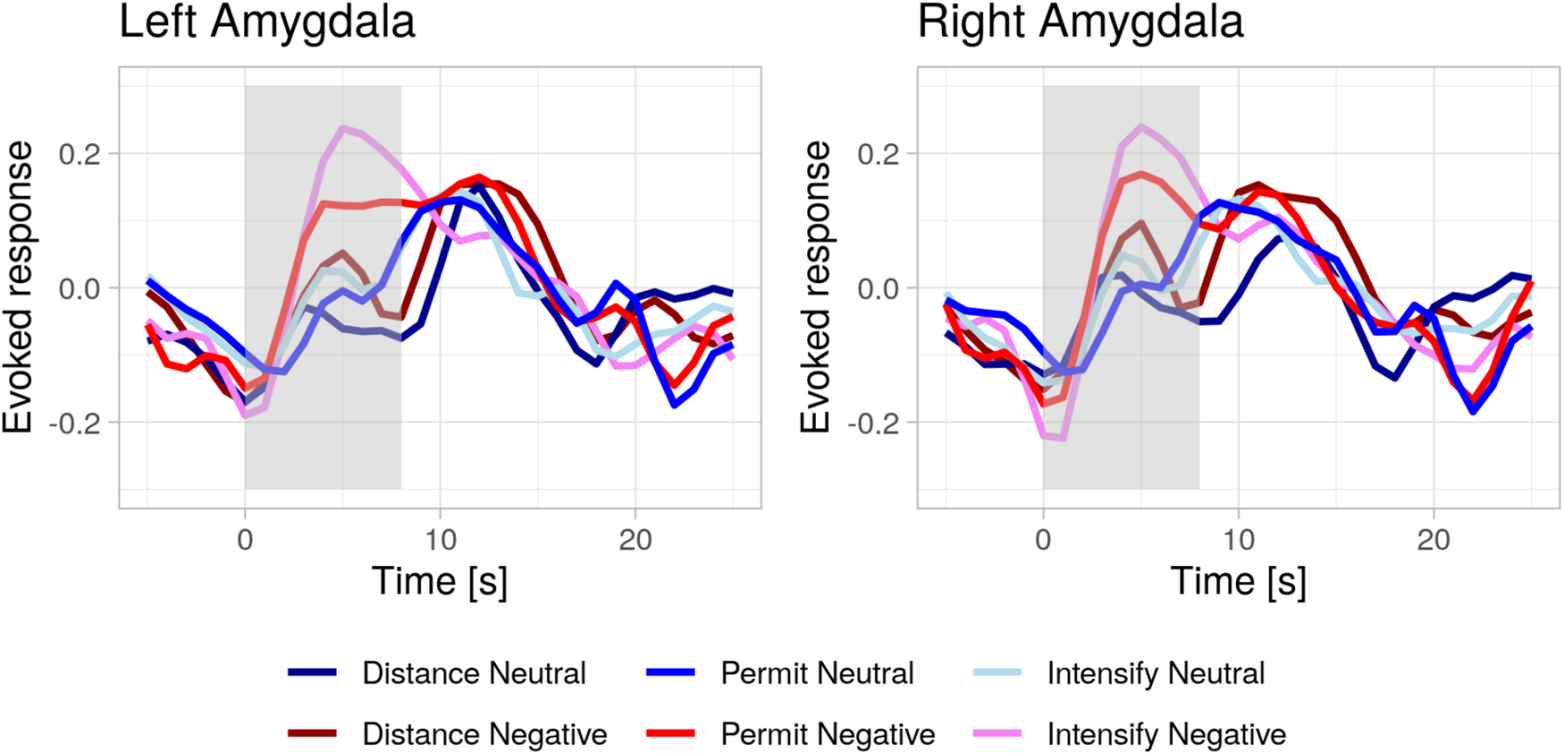
Activation time courses in the left and right amygdala. The shaded area indicates the stimulation phase. Time courses were determined for every participant by means of a finite impulse response (FIR) model for the regressors of each experimental condition, and subsequently averaged across participants. Values on the y-axis reflect the beta coefficients of the FIR model.

An additional aspect of these data was revealed by a post-hoc analysis of the temporal course of activation for the left and right amygdala (Fig. 5). This descriptive analysis shows transient peaks in amygdala activation at onset and again at offset of the stimulation phase.

### 3.4 Activation differences during re-exposure (timepoints 3 and 4)

We finally report differences between previously regulated and non-regulated items during re-exposure after 10 min and after 1 week.

#### 3.4.1 Re-exposure after 10 min (timepoint 3)

**Fig. 6.**
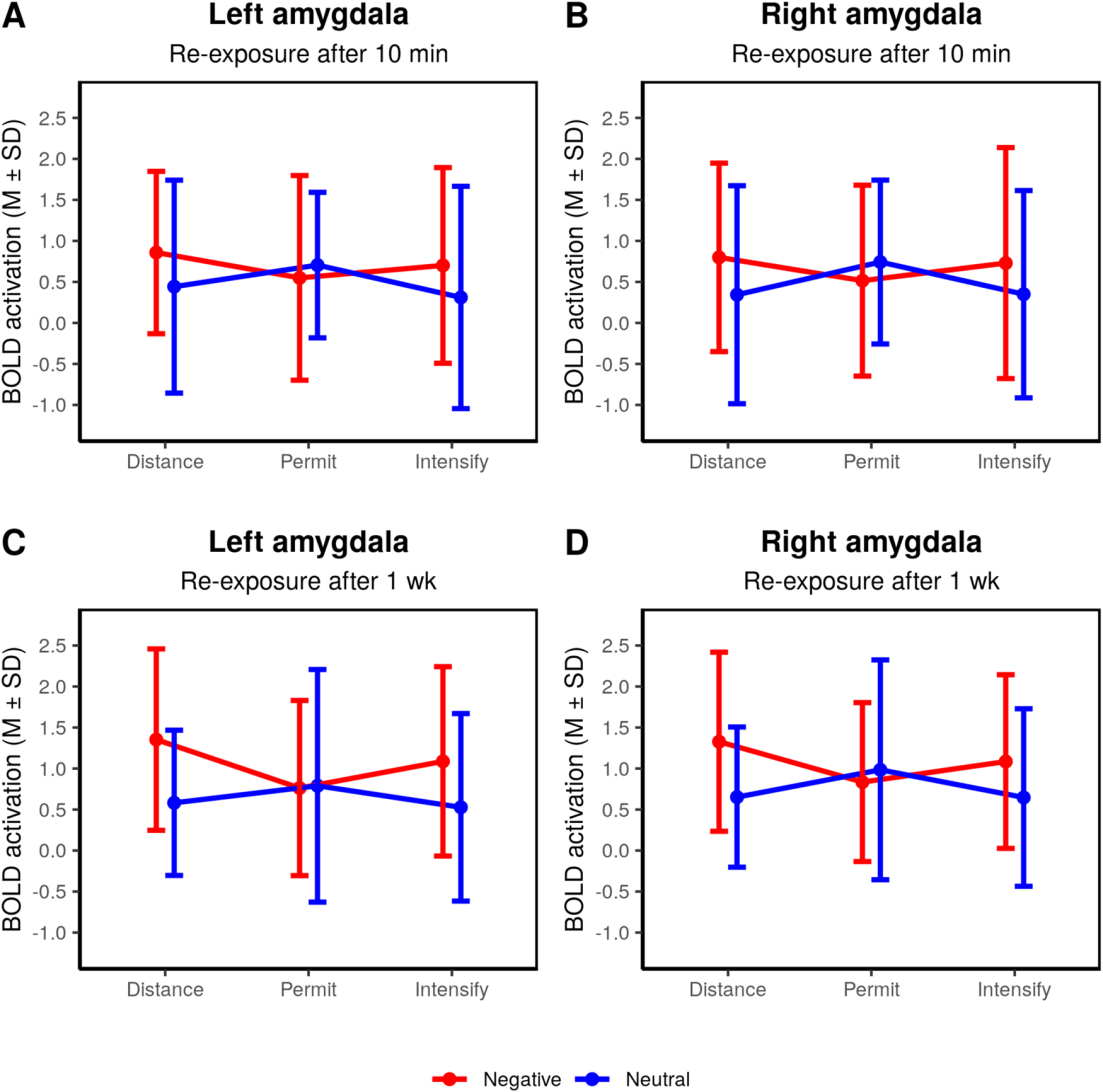
Summary statistics for the amygdala ROI during re-exposure after 10 min and after 1 week. Lines represent mean and standard deviations for each condition.

During re-exposure after 10 min, we observed a main effect of “picture” within the left and right temporal and occipital cortices, but not the left or right amygdala ROI (Table 3A). No other effect was significant in this analysis; in particular, the mode of regulation during the main experiment did not impact the activation in response to the stimuli when they were presented for a second time, neither for the overall analysis, nor for separate analyses or negative or neutral stimuli, respectively (Table S4A and S4B). We observed no significant effects in either the left amygdala (Picture: F(1,40)=2.83, p=.100, $\eta^{2}=.009$; Regulation: F(2,80)=0.47, p=.630, $\eta^{2}=.003$), Picture × Regulation: F(2,80)=1.65, p=.198, $\eta^{2}=.013$) or the right amygdala (Picture: F(1,40)=1.39, p=.245, $\eta^{2}=.007$; Regulation: F(2,80)=0.13, p=.876, $\eta^{2}=.001$; Picture × Regulation: F(2,80)=2.17, p=.122, $\eta^{2}=.016$; Fig. 6A and 6B). Pairwise comparisons are reported in Table S8.

**Table 3.** Activation maxima during re-exposure runs after 10 min (section A) and 1 week (section B).

A. RE-EXPOSURE AFTER 10 MIN (ALL STIMULI)							
K	*p_FWE_*	*t*	*p_unc._*	x	y	z	Label
Main effect Picture							
419	*<*0.001	43.22	*<*0.001	52	-74	-4	Right Inferior Temporal Gyrus
33	0.002	30.72	*<*0.001	-64	-24	2	Left Middle Temporal Gyrus
101	0.004	29.00	*<*0.001	-42	-82	4	Left Middle Occipital Gyrus
91	0.005	28.45	*<*0.001	58	-8	4	Right Superior Temporal Gyrus
76	0.006	28.13	*<*0.001	42	-62	-12	Right Inferior Occipital Gyrus
30	0.007	27.96	*<*0.001	-40	-52	-18	Left Fusiform Gyrus
Main effect Regulation							
— *No results* —							
Interaction effect Picture x Regulation							
— *No results* —							
Distance *>* Permit							
— *No results* —							
Permit *>* Distance							
— *No results* —							
Permit *>* Intensify							
— *No results* —							
Intensify *>* Permit							
— *No results* —							
Intensify *>* Distance							
— *No results* —							
Distance *>* Intensify							
— *No results* —							
(DistanceNeutral *>* PermitNeutral) *>* (DistanceNegative *>* PermitNegative)							
— *No results* —							
(PermitNeutral *>* DistanceNeutral) *>* (PermitNegative *>* DistanceNegative)							
— *No results* —							
(PermitNeutral *>* IntensifyNeutral) *>* (PermitNegative *>* IntensifyNegative)							
— *No results* —							
(IntensifyNeutral *>* PermitNeutral) *>* (IntensifyNegative *>* PermitNegative)							
— *No results* —							
(IntensifyNeutral *>* DistanceNeutral) *>* (IntensifyNegative *>* DistanceNegative)							
— *No results* —							
(DistanceNeutral *>* IntensifyNeutral) *>* (DistanceNegative *>* IntensifyNegative)							
— *No results* —							

B. RE-EXPOSURE AFTER 1 WEEK (ALL STIMULI)							
K	*p_FWE_*	*t*	*p_unc._*	x	y	z	Label
Main effect Picture							
3115	*<*0.001	79.03	*<*0.001	-40	-56	-18	Left Fusiform Gyrus
3775	*<*0.001	73.90	*<*0.001	44	-76	-12	Right Inferior Occipital Gyrus
179	*<*0.001	42.79	*<*0.001	30	-56	50	Right Inferior Parietal Lobule
18	0.007	14.12	*<*0.001	-18	-6	-16	Left Amygdala (ROI)
Main effect Regulation							
— *No results* —							
Interaction effect Picture x Regulation							
— *No results* —							
Distance *>* Permit							
— *No results* —							
Permit *>* Distance							
— *No results* —							
Permit *>* Intensify							
— *No results* —							
Intensify *>* Permit							
— *No results* —							
Intensify *>* Distance							
— *No results* —							
Distance *>* Intensify							
— *No results* —							
(DistanceNeutral *>* PermitNeutral) *>* (DistanceNegative *>* PermitNegative)							
— *No results* —							
(PermitNeutral *>* DistanceNeutral) *>* (PermitNegative *>* DistanceNegative)							
— *No results* —							
(PermitNeutral *>* IntensifyNeutral) *>* (PermitNegative *>* IntensifyNegative)							
— *No results* —							
(IntensifyNeutral *>* PermitNeutral) *>* (IntensifyNegative *>* PermitNegative)							
— *No results* —							
(IntensifyNeutral *>* DistanceNeutral) *>* (IntensifyNegative *>* DistanceNegative)							
— *No results* —							
(DistanceNeutral *>* IntensifyNeutral) *>* (DistanceNegative *>* IntensifyNegative)							
— *No results* —							

Abbreviations as in Table 1.

#### 3.4.2 Re-exposure after 1 week (timepoint 4)

A similar pattern emerged for the re-exposure after 1 week (Table 3B). Here, we also observed the main effects of “picture,” in this case in the left fusiform gyrus, the right occipital and inferior parietal cortex, as well as the left amygdala. However, no other effects were present. In particular, previous emotion regulation did not influence the activation in response to the pictures after 1 week, neither for the overall analysis, nor for separate analyses or negative or neutral stimuli (Table S4C and S4D). Furthermore, we observed significant “picture” effects in the left and right amygdala (left amygdala: F(1,29)=8.76, p=.006, $\eta^{2}=.036$; right amygdala: F(1,29)=4.73, p=.038, $\eta^{2}=.022$). However, no regulation effects were present (left amygdala: F(2,58)=0.77, p=.466, $\eta^{2}=.006$; right amygdala: F(2,58)=0.29, p=.748, $\eta^{2}=.002$). A “picture” × “regulation” interaction effect was only present in the right amygdala (left amygdala: F(2,58)=2.93, p=.061, $\eta^{2}=.022$; right amygdala: F(2,58)=3.32, p=.043, $\eta^{2}=.026$). Figure 6C and 6D indicates, on average, greater activation for the negative in comparison to the neutral stimuli; this effect was more pronounced for the items that were previously regulated, that is, presented during the distance or intensify condition. Pairwise comparisons are reported in Table S8.

## 4 Discussion

This study presented an investigation of the neural basis of up- and down-regulation of negative and neutral stimuli at immediate as well as short- and long-term delays. Its main results can be summarized as follows: first, greater activation in emotion-generating regions such as the amygdala was observed in the permit vs. distance and the intensify vs. distance comparisons, but not in the intensify vs. permit comparison. Second, greater activation in emotion-regulating regions such as the right inferior parietal and right superior / middle frontal cortex activation was found in the distance vs. permit and the distance vs. intensify contrasts. Third, the activation difference between distance and intensify within the amygdala reversed after the regulation period. Fourth, previous emotion regulation did not influence the activation in response to the pictures, neither after 10 min nor after 1 week.

Taken together, the current study confirms and extends the present knowledge about the neural bases of cognitive emotion regulation: first of all, it corroborates the evidence for the general efficacy of cognitive emotion regulation paradigms. This primarily concerns the decrease of amygdala activation with a concurrent increase of activation in frontal and parietal regions during down-regulation. This finding is in line with results from numerous single studies (Dörfel et al., 2014; Kanske, Heissler, Schönfelder, Bongers, & Wessa, 2011) as well as several reviews and meta-analyses (Frank et al., 2014; Morawetz et al., 2017; Ochsner et al., 2012). Next, we were able to directly compare the activation patterns during down-regulation with those during an up-regulation condition. Only the left supplementary motor area and the precentral gyrus were involved in both up- and down-regulation of emotion. We also found activation specific to the distance condition in the right frontal and parietal cortex, and specific to the intensify condition in the left frontal and precentral cortex. This observation not only indicates that up- and down-regulation could rely on different brain regions, but also that the frontal and parietal regions, which are commonly implicated in emotion-regulation, may only be involved in the down- but not the up-regulation of emotions. Further, it is noteworthy that several effects such as the right middle frontal gyrus only appeared in the distance vs. intensify comparisons, but not in the comparisons of either condition against the permit condition. This suggests that adding an up-regulation condition to the experimental paradigm has a beneficial effect as some contrasts appear to require a maximization of differences between the experimental conditions. In addition to the particular conditions, their systematic combination within a full factorial design allowed us not only to assess their main effects, but also possible interaction effects of the “picture” and “regulation” factors. In the voxel-wise analyses, we did not observe any interaction effects between “picture” and “regulation.” This points to a rather domain-general role of regulation effects irrespective of picture valence, that is, comparable implementation of regulation strategies across stimulus categories. An exception of this interpretation is the amygdala, where we observed interaction effects in the analysis of summary statistics, but not in the voxel-wise analyses. In this region, which we consider the target of top-down emotion regulation, our analysis remains inconclusive with regard to domain-specific or domain-general regulation effects.

Finally, this study distinguished between immediate, short- and long-term regulation effects. Considering the immediate effects, we confirm the importance of the temporal analysis model for the detection of subtle activation effects; the detection of amygdala activation, in particular, crucially depended on a suitable analysis model, in this case a model for transient responses. We also confirm that there is substantial variability within an experimental trial, that is, during and also after the stimulation phase. An example of this variability is the so-called rebound effect, which was originally reported as a paradoxical increase of amygdala activation after preceding down-regulation (Walter et al., 2009). Here, we studied this effect in a more complex context; we found a reversal of activation differences between the distance and intensify conditions, but this did not appear as clear-cut as in the original study. Again, we could detect this effect only using a transient response model. A less stringent pattern emerged for the more delayed time windows; after 10 min and 1 week, we were not able to detect an influence of the preceding regulation efforts. There are several implications of these results; in the remainder, we will focus on the comparison between up- and down-regulation, the temporal effects, and the replication of the results of our previous study.

### 4.1 Comparison of up- vs. down-regulation

The first goal of this study has been to determine if up- and down-regulation rely on same or different neural systems. Conceptually, this touches the question whether or not there is a general capacity for emotional regulation. Such a capacity would imply that the direction of regulation is less important than the particular process that is employed to achieve the desired effect—reinterpretation, for example, could be used for both up- and down-regulation. If down-regulation strategies had such up-regulation counterparts, this would support the idea that also the same brain regions are involved. Conversely, segregated activation would rather support an independence model.

The results of this study speak against the assumption of a common regulation system. The primary activation foci in the distance condition were the right angular gyrus and the right superior frontal gyrus. The intensify condition, in contrast, primarily yielded activation in the left precentral, frontal, and occipital areas. Activation in the right angular gyrus even decreased. Further, there were several regions that showed clear differences when the intensify and distance conditions were directly contrasted with each other; among these are right inferior parietal cortex, right middle frontal gyrus, left angular gyrus and the precuneus (all distance > intensify), and the amygdala and the occipital cortex (both intensify > distance). These observations are somewhat discrepant to the results of Min et al. (2022), who found common activation in, for example, the inferior frontal gyrus and dorsal anterior cingulate gyrus during up- and down-regulation, but modulation of distinct emotion-generating during either mode of regulation. While our results are more in line with the traditional affective-dial hypothesis, differences between the two studies might be explained by different cortical regions found during up- and down-regulation, potential differences in the implementation of regulation strategies, or lesser statistical power to detect such effects in our study.

Taken together, we observe largely non-overlapping activation patterns that are additionally characterized by differences in lateralization—no clear lateralization for intensify, but predominant right-hemispheric activation for distance. Similar effects have been reported in the literature, for example, left/right lateralization of prefrontal activation for up- and down-regulation (Kim & Hamann, 2007), although there are also reports of largely overlapping activation patterns (Eippert et al., 2007). Our results also concur with the available meta-analytic evidence, in particular with regard to the activation uniquely related to down-regulation (Morawetz et al., 2017), while there is only partial congruence with respect to up-regulation or common regulation effects.

An implication of independent regulation strategies is that the distance vs. permit contrast and the intensify vs. permit contrast will yield non-overlapping sets of differently activated regions. From this follows that if a given region—such as the right superior frontal gyrus in our case—exhibits an effect of the distance condition, this does not necessarily mean that there is also an intensify vs. permit difference in this particular region. In that sense, a different assumption than the independence model is a quasi-linear increase—or decrease—of activation in the same region from the distance via the permit to the intensify condition. Indeed, we found such patterns, for example, in the right inferior parietal lobe, which showed greater activation during distance than during permit, and during permit than during intensify. The reverse pattern, that is, greater activation during intensify as compared to permit, and during permit as compared to distance, occurs in the middle occipital gyrus. On the other hand, patterns such as u- or inverted u-like relations, which would support a general regulation effect, were not observed. The finding of primarily monotonically increasing or decreasing patterns suggests that there is not necessarily independence, nor a general regulation effect, but rather an inverse relationship between the distance and intensify condition—and interestingly, not only on a subcortical level in the emotion-generating regions, but also—with the opposite direction—in the cortex.

The observation of segregated activation patterns in the two regulation conditions also suggests that different cognitive processes support these kinds of regulation. In fact, our experimental conditions were only moderately similar. Although both are related to the concepts of approaching or distancing, they could be implemented in very different ways; intensify could be achieved by increasing one’s personal or physical involvement with the given situation, whereas distancing could be achieved by taking the position of a non-involved observer, by reducing the personal involvement, or by assuming a personal or physical distance. It is obvious that this leads to considerable heterogeneity; in this regard, narrowing the available regulation strategies down to a more circumscribed set of cognitive processes might have led to less heterogeneous, more comparable patterns of neural activation.

### 4.2 Temporal aspects of up-regulation

The second major goal of this study has been to describe the effects of the up- and down-regulation not only with regard to their spatial distribution, but also from the point of view of distinct temporal activation patterns.

Considering the activation dynamics within a single a experimental trial, we propose that the transient responses model is better suited to detect amygdala activation than the sustained responses model. This applies to both the regulation-related as well as the post-stimulation activation. The rebound effect in particular depends on the statistical model; it was present for the transient but not the sustained responses. Further, the detection of the rebound effect also depends on the particular condition under consideration; it was most pronounced for the intensify and distance conditions. We therefore conclude that it is beneficial to maximize the contrast between any two regulation conditions for the investigation of regulation aftereffects. Altogether, a new contribution of this study is the observation that aftereffects are not only observable during a comparison of a regulating and a non-regulation condition (such as distance and permit), but also observable during a contrast of the down- vs. the up-regulation of emotion (i.e, distance and intensify).

Similar to previous studies, we observe temporal effects also in regions beyond the amygdala. Again, these effects are heterogeneous: in temporal and occipital regions, we observe similar patterns as in the amygdala, that is, in all of these regions, the activation increases during intensify (vs. distance) in the stimulation phase reversed during the post-stimulation phase. This may not be surprising, given our observation that occipital and temporal co-activate with the amygdala during the stimulation phase. The reverse effect was observed in the right inferior parietal lobe; this may indicate that opposite patterns from the stimulation phase continue into the post-stimulation phase, similar to the observation of concordant task-rest interactions in Lamke et al. (2014).

An unexpected finding, at least in the light of previous results, was the missing re-exposure effect after 10 min in the amygdala, since we did not observe a difference in activation between negative and neutral stimuli. This is surprising given the large body of results regarding the role of the amygdala in the processing of arousing vs. non-arousing emotional material. We speculate that this may reflect a certain satiation effect, considering that the previous presentation of the same material was still very recent. Consistent with this explanation is the observation that after 1 week the expected difference between the two stimulus conditions was again present. Apart from the discrepant results regarding the stimulus effects, we did not observe any lasting effect of previous regulation efforts. This means that neither the down- nor the up-regulation condition did have an effect on amygdala activation at later stage. This speaks against the long-term efficacy of the regulation conditions as implemented in the current experimental paradigm.

### 4.3 Replication and extension of Diers et al. (2021)

Our third and final goal has been to relate the current findings to the results of our previous study (Diers et al., 2021). The two studies share the same experimental design, with the major difference that we added the intensification condition in the present study. This led to minor adjustments in the number and duration of stimulus presentations, and a slightly increased overall duration of the experiment. Apart from that, the main design characteristics were the same, in particular the distance and permit conditions, the slow event-related design, and the re-exposure sessions after 10 min and after 1 week. This overall similarity allows for a replication, but also for answering questions beyond replication.

The main results of our previous study were activation in the right middle frontal and inferior parietal cortex during distancing while concurrent amygdala activation was decreased. The cortical effects primarily appeared as a sustained response, whereas the amygdala exhibited a pattern of transient responses. Paradoxical aftereffects were observed in the amygdala, the occipital cortex, and the ventromedial frontal / subgenual cingulate cortex. Finally, previous emotion regulation led to an increase of amygdala activation during the re-exposure sessions. A comparison of these results with our current ones supports the following conclusions: a considerable overlap between the down-regulation activation patterns—both experiments involve the right inferior parietal and right middle frontal gyrus—suggests that down-regulation of the amygdala is a reliable effect, and further in bidirectional settings works similar to unidirectional settings. Both studies also provided limited support for the rebound effect.

Apart from these commonalities, we also observed discrepant results between the two studies. This primarily concerns the effect of previous emotion regulation on amygdala activation during re-exposure. In contrast to the first study, no such effect was present in the second study. A potential explanation of this result is that a delayed regulation effect in a passive viewing task would require some kind of incidental, automated processing. Given the increased complexity of the task in our current study, it might have been more difficult to acquire such automated processing in the current experimental context of two, instead of one, regulation conditions.

The comparison between our two experiments also gives rise to a more general question regarding the consistency and comparability of conditions across experiments. Specifically, are the distance and permit conditions the same in the two experiments? Or do participants internally strive to maximize contrast between tasks, which might have led to an unintentional—and undetectable—up-regulation of emotion during the permit condition in the previous study. An indication of this might be the observation of middle frontal activation in the distance vs. intensify contrast in the current experiment vs. the observation of the same activation in the distance vs. permit contrast in the previous experiment. In other words, do experimental conditions in regulation paradigms depend on the particular framing and context? If so, this might account for the following observations: first, no activation difference between permit and intensify in study 2; second, approximately linear activation increases or decreases across regulation conditions in study 2; third, the partial similarity of the permit > distance contrast in study 1 and the intensify > distance contrast in study 2, which both led to amygdala and occipital activation, although in study 2 we additionally observed left precentral and frontal activation. This particular observation of unique intensify-related activation in higher cortical areas, however, speaks against the context-dependence of our experimental conditions.

### 4.4 Limitations

Two general limitations of the current study are that we were not able to investigate the impact of positive valence, and that we had to restrict the design to exactly one up- and one down-regulation condition. Both limitations result from the need of limiting the duration and complexity of the task, but nevertheless they prevent a comparative analysis of stimulus characteristics and regulation strategies. It is well possible that emotional regulation works differently for positive and negative stimuli, or that alternative down- and up-regulation strategies prove either more or less effective or more or less similar to each other than the intensify and distance conditions that we used. These are questions that need to be addressed by other studies of a similar experimental design.

In addition, there are a few other issues that restrict our conclusions. First, we cannot entirely be sure about the success of the intensify condition. This is due to the missing increase of amygdala activation as compared to the permit condition. For exploratory reasons, we investigated post-hoc ratings on perceived confidence and difficulty in applying the three emotion regulation strategies (see Figs. S4 and S5). Descriptive results indicate that the permit and intensify conditions were perceived as similar to each other with respect to difficulty and confidence, but as different compared to the distance condition. However, this does not necessarily imply that participants did not up-regulate their emotions, given that there was a contrast with the distance condition in amygdala activation, and also unique frontal activation in the up-regulation condition. It is possible that the three conditions—each one associated with its own specific instructions—already pose too much complexity to the participants so that they resorted to internally simplifying the tasks. It may also be that intensification is simply not effective enough. In all cases, it would be helpful to employ alternative, and possibly less demanding strategies.

A second issue concerns the fact that we did not observe any short- or long-term transfer of previous emotion regulation to the re-exposure sessions. This is only partially consistent with our observations in the first study. One reason might be that—in spite of a more complex task—we possibly did not allow for enough training to enable successful transfer. This would call for a higher number of experimental trials or repeated presentations of the same stimuli during the stimulation phase, allowing for practicing stimulus-specific regulation strategies. Unrelated to this is the question whether or not emotion regulation can be practiced at all, and whether the acquired skills also generalize to previously unseen stimuli. Both remain questions for further research.

Third, the study participants were young and healthy university students. While this is not a limitation of the validity of the present findings, the generalizability to other healthy and clinical populations and age cohorts is nevertheless constrained and should be investigated in further research.

A final issue arises from the attrition of participants in the follow-up measurement after 1 week, for which only about two thirds of the participants returned. While we can only speculate about the reasons, the implications are two-fold: first, the reduced sample size leads to a decrease in statistical power. This means that some of our null results for the follow-up measurements could possibly be due to limited ability to detect potential differences between the experimental conditions. Second, the missing data for the follow-up measurement may have occurred in a non-random fashion. That is, demographic or other personal characteristics might differ between persons who returned and those who did not (although sex, age, and arousal ratings did not differ between the first and the follow-up measurement; see Fig. S6). As a consequence, we cannot rule out a bias with regard to participant characteristics in these analyses.

## 5 Conclusion

The goal of this study has been to contrast emotional up- and down-regulation at immediate, and short- and long-term delays. To this end, we employed three experimental conditions—distance, permit, and intensify—for negative and neutral stimuli, and investigated the neural responses during the regulation and post-stimulation phase as well as after 10 min and after 1 week. We found that each emotional regulation condition has distinct neural signatures, and exhibits distinct temporal dynamics. We thus replicate and extend several results of our previous study. Although some issues such as the temporal aspects still call for further research and replication, it is hoped that this has opened up, as stated at the beginning, a broader perspective on emotion regulation that should not exclusively be understood as the down-regulation of negative emotion.

## Supplementary Material

Supplementary Material

## Data Availability

The dataset analyzed for this study as well as the analysis code and materials are openly available at the Open Science Framework (https://osf.io/ktjnw). This study was not preregistered.
