# Supplementary material for: Short- and long-term effects of emotion up- and down-regulation
Source: Imaging Neurosci (Camb). 2023 Nov 9;1:imag-1-00028. doi: 10.1162/imag_a_00028 (PMC12007535; doi:10.1162/imag_a_00028)
Supplement: Supplementary Material [file imag_a_00028-supp.pdf]

Short- and long-term effects of emotion up- and down-regulation

Supplemental material

Kersten Diers*[1], Anne Gärtner*[1], Sabine Schönfeld[2], Denise Dörfel[1], Henrik Walter[3], Burkhard Brocke[1], and Alexander Strobel[1]

[1]Faculty of Psychology, Technische Universität Dresden, Dresden, Germany

[2]Evangelische Hochschule Dresden, Dresden, Germany

[3]Division of Mind and Brain Research, Department of Psychiatry and Psychotherapy, CCM, Charité Universitätsmedizin, Berlin, Germany

Corresponding Author:

Anne Gärtner, PhD

Faculty of Psychology

Technische Universität Dresden

01069 Dresden, Germany

Phone: +49-351-463-36997

Email: anne_gaertner@tu-dresden.de

*These authors contributed equally.

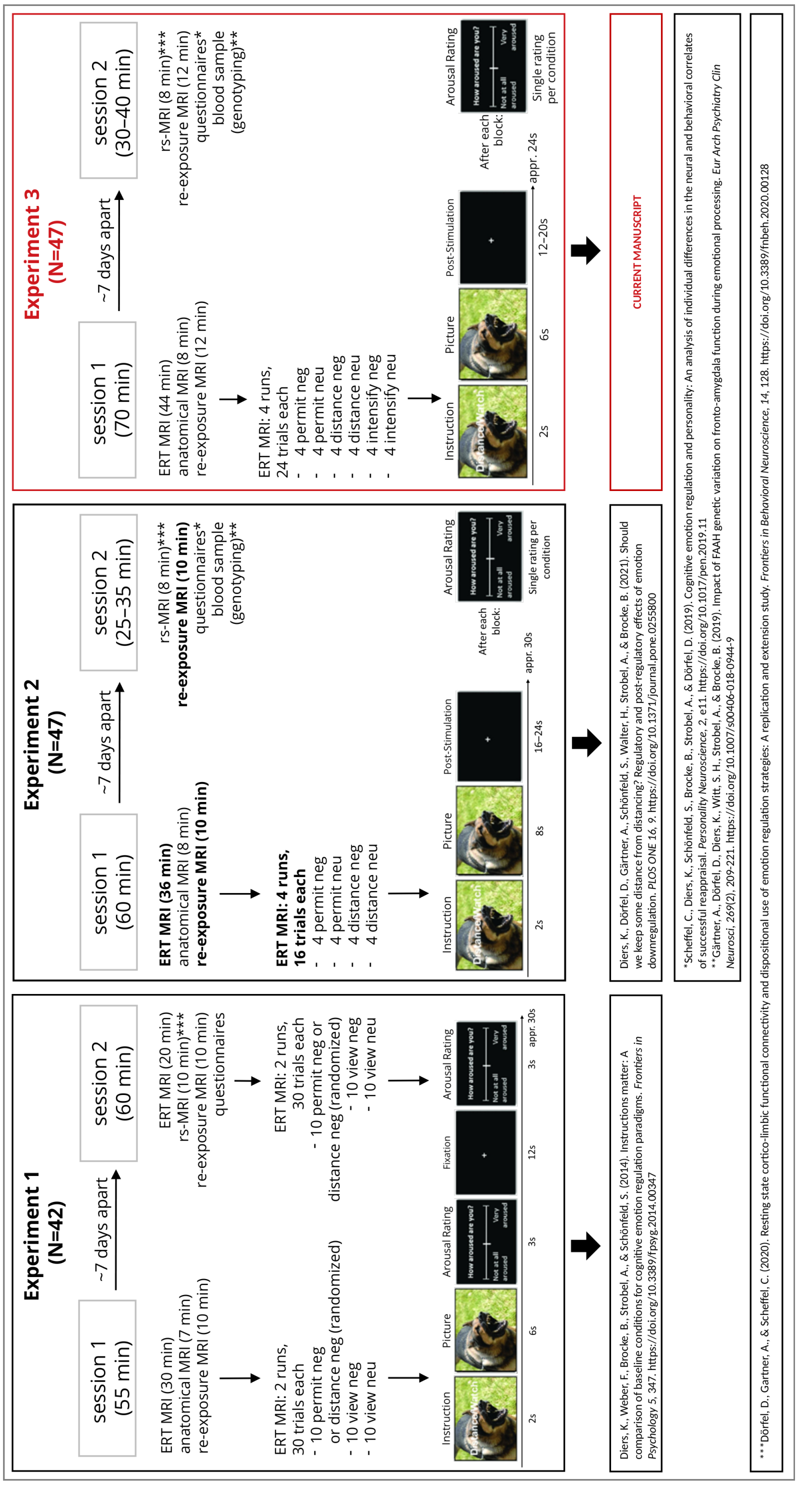

Figure S1. Overview over the larger project and corresponding peer-reviewed publications and manuscripts, respectively. The present manuscript is highlighted in red and parts of the experimental procedure relevant for the present study in bold letters. ERT = Emotion Regulation Task, rs-MRI = resting-state MRI, neg = negative pictures, neu = neutral pictures.

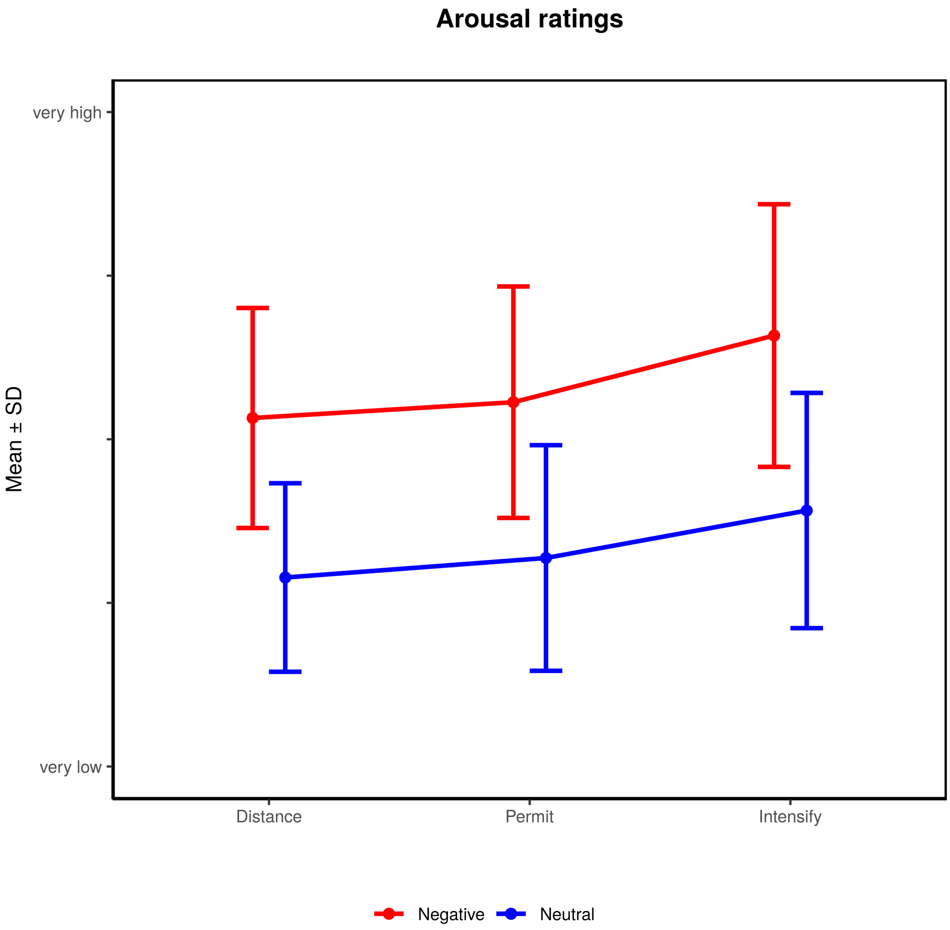

Figure S2. Mean and standard deviations for subjective arousal ratings during the emotion regulation task.

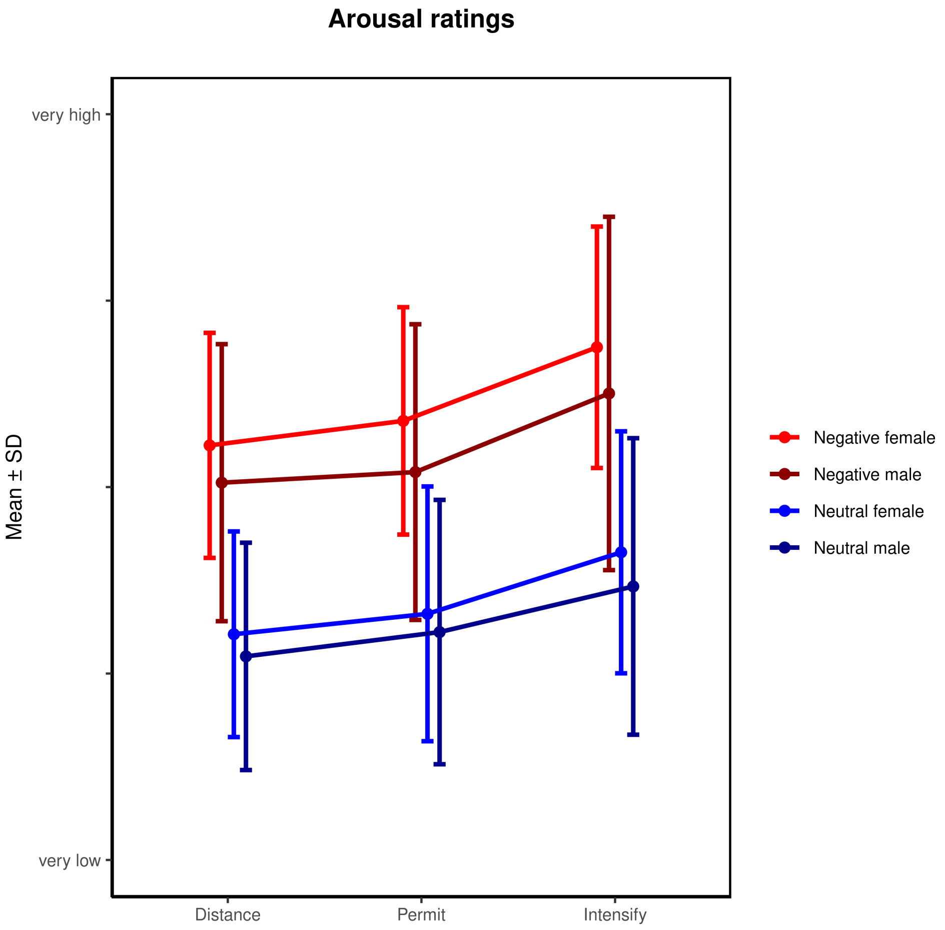

Figure S3. Mean and standard deviations for subjective arousal ratings during the emotion regulation task, separated by sex.

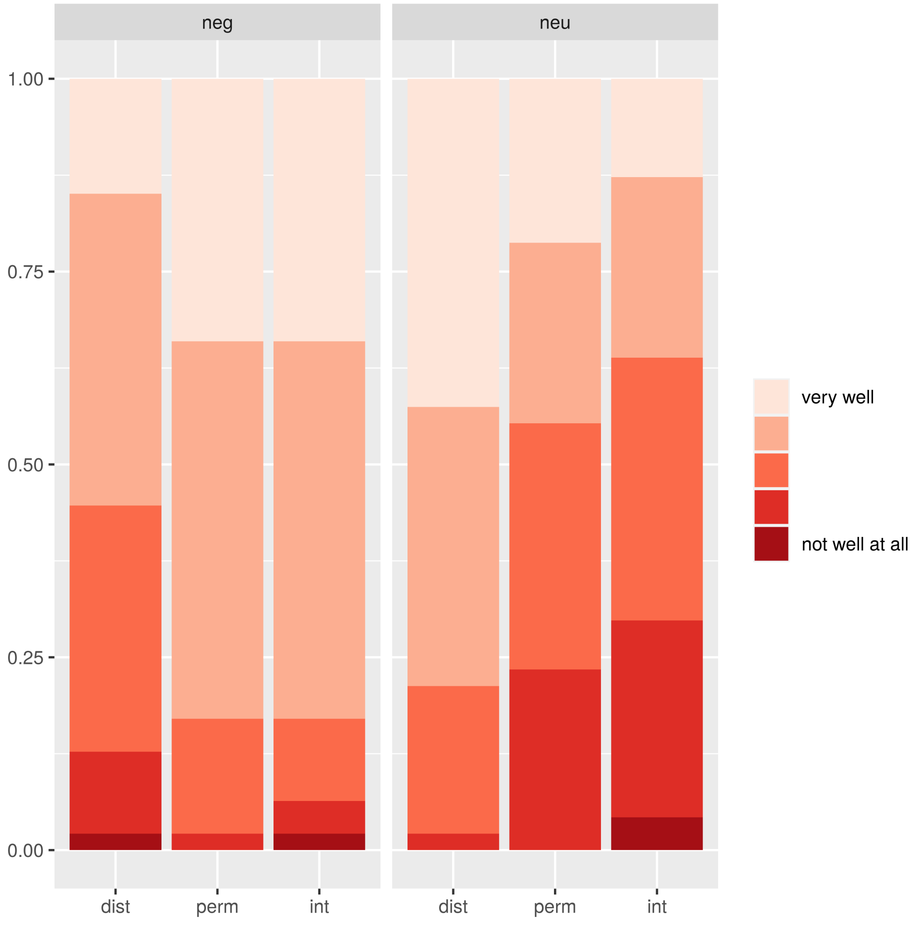

Figure S4. Post-experiment ratings in response to the question **"Please indicate how well you could implement the instruction"** for the distance, permit, and intensify instruction, and for negative and neutral pictures. The y-axis represents proportions of participants. Abbreviations: dist=distance, perm=permit, int=intensify; neg=negative, neu=neutral.

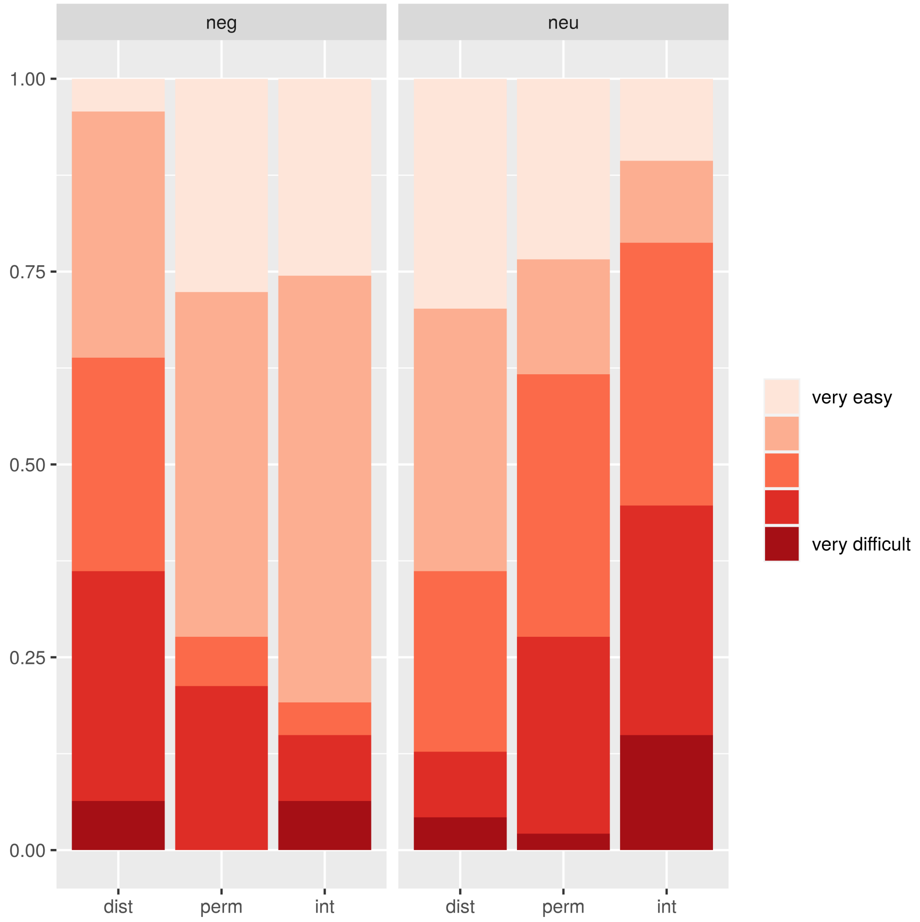

Figure S5. Post-experiment ratings in response to the question **“Please indicate how difficult it was to implement the instruction”** for the distance, permit, and intensify instruction, and for negative and neutral pictures. The y-axis represents proportions of participants. Abbreviations: dist=distance, perm=permit, int=intensify; neg=negative, neu=neutral.

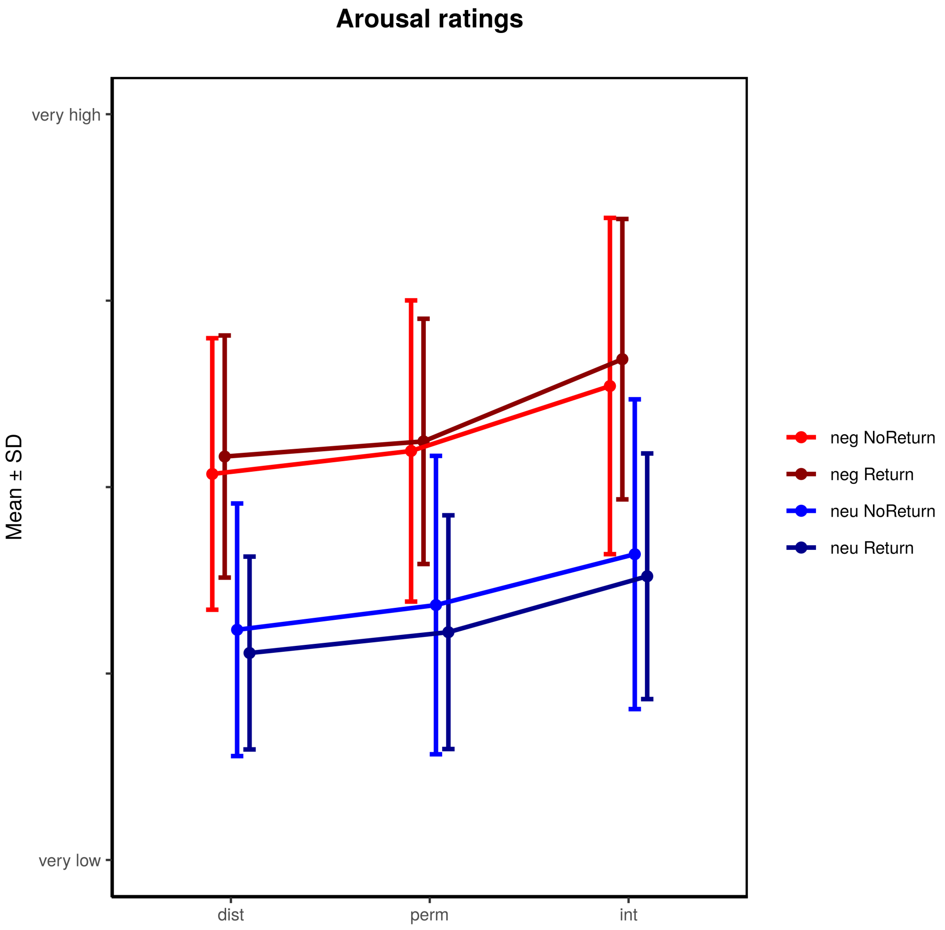

Figure S6. Mean and standard deviations for subjective arousal ratings during the emotion regulation task for participants who returned for the re-exposure after 1 week and those who did not. Abbreviations: dist=distance, perm=permit, int=intensify; neg=negative, neu=neutral.

In a comparison of persons who returned after one week and those who did not, we found no demographic differences nor differences in the arousal ratings (cf. Figure S6). Specifically, both groups showed a similar ratio of male/female participants (53.3% female in the group of participants who returned, 52.9% female in the group who did not return), and did not differ in their mean age (24.87 years for participants who returned, 25.88 years for participants who did not return; $t = 0.72$, $p = 0.48$). Furthermore, we did not observe any effects of returning or not on the arousal ratings (main effect of Returning: $F = 0.001, p = 0.929$, interaction of Returning and Picture: $F = 1.503$, $p = 0.227$, interaction of Returning and Regulation: $F = 0.252$, $p = 0.778$, interaction of Returning, Picture and Regulation: $F = 0.268$, $p = 0.766$), and the observed main and interaction effects for the "picture" and "regulation" factors remained unchanged when controlling for return status (main effect of Picture: $F = 110.404$, $p < 0.001$, main effect of Regulation: $F = 62.514$, $p < 0.001$, interaction of Picture and Regulation: $F = 3.192$, $p = 0.046$).

Table S1. Activation maxima during the emotion regulation task. Reported are results during the stimulation phase. Abbreviations: k = spatial extent, p_{FWE} = p-values corrected for multiple comparisons (FWE), $p_{unc.}$ = uncorrected p-values, t = t-statistics, x, y, z = MNI coordinates. ROI indicates that an activation peak was observed within the left or right amygdala region of interest.

k	p_{FWE}	t	$p_{unc.}$	x	y	z	Label
**STIMULATION PHASE (ALL STIMULI)**							
**Main effect Picture**							
16721	<0.001	187.85	<0.001	-46	-80	0	Left Middle Occipital Gyrus
666	<0.001	49.80	<0.001	44	8	30	RightPrecentral Gyrus
1465	<0.001	47.43	<0.001	10	-14	50	Right Middle Cingulate Cortex
567	<0.001	43.85	<0.001	58	-8	0	Right Superior Temporal Gyrus
132	<0.001	42.54	<0.001	2	-32	-4	N/A
221	<0.001	37.85	<0.001	40	-16	18	Right Rolandic Operculum
150	<0.001	34.15	<0.001	-42	-60	52	Left Inferior Parietal Lobule
302	0.001	33.38	<0.001	-34	-28	48	Left Postcentral Gyrus
43	0.003	29.35	<0.001	-26	-48	46	Left Inferior Parietal Lobule
34	0.005	28.61	<0.001	6	52	20	Right Superior Medial Gyrus
148	0.006	28.01	<0.001	-54	-18	10	Left Superior Temporal Gyrus
44	0.009	26.86	<0.001	-38	-20	18	Left Rolandic Operculum
26	0.010	26.74	<0.001	-44	-32	8	Left Superior Temporal Gyrus
90	<0.001	22.53	<0.001	-20	-6	-14	Left Amygdala (ROI, sustained)
97	<0.001	28.10	<0.001	22	-6	-12	Right Amygdala (ROI, sustained)
186	<0.001	59.67	<0.001	-18	-6	-14	Left Amygdala (ROI, transient)
190	<0.001	55.80	<0.001	20	-4	-14	Right Amygdala (ROI, transient)
**Main effect Regulation**							
1798	<0.001	60.94	<0.001	58	-54	40	Right Inferior Parietal Lobule
4034	<0.001	53.26	<0.001	30	-94	0	Right Middle Occipital Gyrus
308	<0.001	22.46	<0.001	44	20	42	Right Middle Frontal Gyrus
262	<0.001	22.15	<0.001	20	14	60	Right Superior Frontal Gyrus
224	<0.001	20.37	<0.001	-8	10	50	Left Supplementary Motor Area
123	<0.001	20.16	<0.001	-4	-24	26	Left Posterior Cingulate Cortex
88	0.001	18.90	<0.001	36	-66	-24	Right Cerebellum
70	0.001	18.43	<0.001	-54	-62	40	Left Angular Gyrus
52	0.002	17.79	<0.001	10	-66	36	Right Precuneus
79	<0.001	12.09	<0.001	-16	-8	-16	Left Amygdala (ROI, sustained)
29	0.011	8.19	<0.001	20	-4	-18	Right Amygdala (ROI, sustained)
194	<0.001	18.60	<0.001	-18	-8	-14	Left Amygdala (ROI, transient)
82	0.001	10.86	<0.001	24	-2	-24	Right Amygdala (ROI, transient)
**Interaction effect Picture x Regulation**							
				— No results —			

Table S2. Activation maxima during the regulation phase of the emotion regulation task (separate analyses for negative [section A] and neutral [section B] stimuli). Abbreviations: k = spatial extent, p_{FWE} = p-values corrected for multiple comparisons (FWE), $p_{unc.}$ = uncorrected p-values, t = t-statistics, x, y, z = MNI coordinates. ROI indicates that an activation peak was observed within the left or right amygdala region of interest.

A. NEGATIVE PICTURES

k	p_{FWE}	t	$p_{unc.}$	x	y	z	Label
**Regulation effect (negative pictures)**							
1223	<0.001	50.39	<0.001	58	-54	44	Right Inferior Parietal Lobule
903	<0.001	39.62	<0.001	30	-94	0	Right Middle Occipital Gyrus
991	<0.001	28.82	<0.001	-30	-92	-8	Left Inferior Occipital Gyrus
135	0.002	17.94	<0.001	42	18	46	Right Middle Frontal Gyrus
33	0.005	16.46	<0.001	-52	-62	44	Left Angular Gyrus
33	0.007	16.07	<0.001	20	24	56	Right Superior Frontal Gyrus
31	0.016	14.95	<0.001	4	-26	26	Right Posterior Cingulate Cortex
13	0.006	8.79	<0.001	-16	-10	-14	Left Amygdala (ROI, sustained)
216	<0.001	19.84	<0.001	-16	-6	-14	Left Amygdala (ROI, transient)
214	<0.001	13.28	<0.001	24	-2	-24	Right Amygdala (ROI, transient)
**DistanceNegative > PermitNegative**							
255	<0.001	6.03	<0.001	54	-50	32	Right Angular Gyrus
**PermitNegative > DistanceNegative**							
635	<0.001	7.72	<0.001	30	-96	-2	Right Inferior Occipital Gyrus
543	<0.001	6.63	<0.001	-30	-92	-6	Left Middle Occipital Gyrus
6	0.014	3.23	0.001	-22	-6	-20	Left Amygdala (ROI, sustained)
103	<0.001	4.27	<0.001	-22	-4	-24	Left Amygdala (ROI, transient)
72	<0.001	4.67	<0.001	24	-2	-24	Right Amygdala (ROI, transient)
**PermitNegative > IntensifyNegative**							
355	<0.001	6.10	<0.001	48	-60	50	Right Angular Gyrus
**IntensifyNegative > PermitNegative**							
58	0.004	5.23	<0.001	0	4	62	Left Supplementary Motor Area
**IntensifyNegative > DistanceNegative**							
2544	<0.001	7.78	<0.001	30	-94	0	Right Middle Occipital Gyrus
44	0.001	4.17	<0.001	-16	-10	-14	Left Amygdala (ROI, sustained)
216	<0.001	6.22	<0.001	-18	-8	-14	Left Amygdala (ROI, transient)
252	<0.001	4.49	<0.001	32	-2	-22	Right Amygdala (ROI, transient)
**DistanceNegative > IntensifyNegative**							
1387	<0.001	10.00	<0.001	58	-54	44	Right Inferior Parietal Lobule
304	<0.001	5.93	<0.001	40	18	46	Right Middle Frontal Gyrus
96	<0.001	5.70	<0.001	-52	-62	44	Left Angular Gyrus
160	0.001	5.60	<0.001	20	22	56	Right Superior Frontal Gyrus
81	0.003	5.26	<0.001	4	-26	26	Right Posterior Cingulate Cortex
70	0.008	5.05	<0.001	44	44	-10	Right Inferior Frontal Gyrus
**(DistanceNegative + IntensifyNegative) > PermitNegative**							
— *No results* —							
**PermitNegative > (DistanceNegative + IntensifyNegative)**							
— *No results* —							

B. NEUTRAL PICTURES

k	p_{FWE}	t	$p_{unc.}$	x	y	z	Label
**Regulation effect (neutral pictures)**							
1321	<0.001	37.33	<0.001	60	-52	36	Right Angular Gyrus
611	<0.001	29.83	<0.001	22	-96	-2	Right Calcarine Gyrus
627	<0.001	19.64	<0.001	-26	-92	4	Left Middle Occipital Gyrus
21	0.008	8.38	<0.001	-16	-10	-16	Left Amygdala (ROI, sustained)
18	0.011	8.25	<0.001	18	-2	-18	Right Amygdala (ROI, sustained)
**DistanceNeutral > PermitNeutral**							
934	<0.001	7.43	<0.001	60	-50	34	Right Angular Gyrus
**PermitNeutral > DistanceNeutral**							
585	<0.001	7.31	<0.001	18	-96	0	Right Calcarine Gyrus

613	<0.001	5.78	<0.001	-32	-88	-4	Left Middle Occipital Gyrus
176	0.002	5.35	<0.001	-10	-78	14	Left Calcarine Gyrus
41	0.007	5.08	<0.001	10	-74	14	Right Calcarine Gyrus
28	0.005	3.56	<0.001	-20	-8	-16	Left Amygdala (ROI, sustained)
61	0.005	3.64	<0.001	20	-4	-18	Right Amygdala (ROI, sustained)
**PermitNeutral > IntensifyNeutral**							
				— No results —			
**IntensifyNeutral > PermitNeutral**							
				— No results —			
**IntensifyNeutral > DistanceNeutral**							
514	<0.001	5.99	<0.001	28	-94	2	Right Middle Occipital Gyrus
164	0.001	5.65	<0.001	-36	-78	-14	Left Fusiform Gyrus
365	0.001	5.50	<0.001	-14	-100	-6	Left Calcarine Gyrus
34	0.004	3.68	<0.001	-16	-10	-16	Left Amygdala (ROI, sustained)
21	0.007	3.52	<0.001	16	-2	-16	Right Amygdala (ROI, sustained)
**DistanceNeutral > IntensifyNeutral**							
1400	<0.001	8.04	<0.001	58	-54	40	Right Inferior Parietal Lobule
62	0.003	5.28	<0.001	8	-66	36	Right Precuneus
31	0.006	5.11	<0.001	44	20	42	Right Middle Frontal Gyrus
**(DistanceNeutral + IntensifyNeutral) > PermitNeutral**							
				— No results —			
**PermitNeutral > (DistanceNeutral + IntensifyNeutral)**							
36	0.007	5.10	<0.001	18	-96	0	Right Calcarine Gyrus

Table S3. Activation maxima during the emotion regulation task. Reported are interaction effects between the stimulation and post-stimulation phase. Abbreviations: k = spatial extent, p_{FWE} = p-values corrected for multiple comparisons (FWE), $p_{unc.}$ = uncorrected p-values, t = t-statistics, x, y, z = MNI coordinates. ROI indicates that an activation peak was observed within the left or right amygdala region of interest.

k	p_{FWE}	t	$p_{unc.}$	x	y	z	Label
**STIMULATION PHASE AND POSTSTIMULATION PHASE (NEGATIVE STIMULI)**							
**Main effect Time**							
30311	<0.001	1690.44	<0.001	-12	-90	-6	Left Calcarine Gyrus
7555	<0.001	229.21	<0.001	22	-28	0	Right Thalamus
4777	<0.001	207.82	<0.001	46	8	28	Right Inferior Frontal Gyrus
17543	<0.001	189.17	<0.001	50	-64	44	Right Angular Gyrus
1262	<0.001	126.76	<0.001	6	12	52	Right Supplementary Motor Area
961	<0.001	116.74	<0.001	-42	-76	40	Left Angular Gyrus
383	<0.001	90.78	<0.001	0	-36	-34	N/A
71	<0.001	65.48	<0.001	-20	-36	-42	Left Cerebellum
1255	<0.001	61.89	<0.001	-16	36	-4	Left Anterior Cingulate Cortex
68	<0.001	55.97	<0.001	22	-36	-42	Right Cerebellum
387	<0.001	45.25	<0.001	22	26	50	Right Superior Frontal Gyrus
172	<0.001	41.32	<0.001	24	-14	62	Right Superior Frontal Gyrus
251	<0.001	35.05	<0.001	-20	32	38	Left Superior Frontal Gyrus
55	<0.001	33.96	<0.001	22	-6	-14	Right Amygdala
46	0.016	25.04	<0.001	-18	-16	60	Left Precentral Gyrus
127	<0.001	24.09	<0.001	-20	-6	-16	Left Amygdala (ROI, sustained)
171	<0.001	33.96	<0.001	22	-6	-14	Right Amygdala (ROI, sustained)
**Main effect Regulation**							
1023	<0.001	27.31	<0.001	54	-54	42	Right Inferior Parietal Lobule
114	<0.001	20.83	<0.001	-34	48	-4	Left Middle Orbital Gyrus
137	0.001	18.07	<0.001	-52	-62	44	Left Angular Gyrus
64	0.002	17.35	<0.001	8	-64	38	Right Precuneus
76	0.002	17.35	<0.001	38	18	48	Right Middle Frontal Gyrus
50	0.006	15.86	<0.001	18	30	52	Right Superior Frontal Gyrus
25	0.010	15.31	<0.001	-4	-68	34	Left Precuneus
48	0.012	15.07	<0.001	-2	-26	28	Left Posterior Cingulate Cortex
**Interaction effect Time x Regulation**							
48	<0.001	22.91	<0.001	60	-52	42	Right Inferior Parietal Lobule
61	0.002	17.15	<0.001	-32	-90	-8	Left Inferior Occipital Gyrus
26	0.012	15.09	<0.001	30	-92	0	Right Middle Occipital Gyrus
32	0.013	14.97	<0.001	0	2	64	Left Supplementary Motor Area
15	0.002	9.67	<0.001	-16	-10	-14	Left Amygdala (ROI, sustained)
173	<0.001	17.50	<0.001	-16	-4	-16	Left Amygdala (ROI, transient)
133	0.001	10.92	<0.001	26	-2	-26	Right Amygdala (ROI, transient)
**Regulation effect (Stimulation phase)**							
1019	<0.001	46.28	<0.001	58	-56	42	Right Inferior Parietal Lobule
126	<0.001	20.21	<0.001	30	-96	-2	Right Inferior Occipital Gyrus
149	<0.001	19.58	<0.001	-28	-96	-6	Left Inferior Occipital Gyrus
49	<0.001	19.45	<0.001	-52	-62	44	Left Angular Gyrus
33	0.009	15.42	<0.001	42	18	46	Right Middle Frontal Gyrus
32	0.010	15.32	<0.001	44	46	-12	Right Inferior Frontal Gyrus
211	<0.001	18.22	<0.001	-16	-8	-16	Left Amygdala (ROI, transient)
208	0.001	11.73	<0.001	26	-2	-24	Right Amygdala (ROI, transient)
**Regulation effect (Post-stimulation phase)**							
169	0.001	18.04	<0.001	-6	-78	30	Left Cuneus
**Distance > Permit**							
				— No results —			
**Permit > Distance**							
				— No results —			
**Permit > Intensify**							
29	0.006	5.08	<0.001	-34	48	-4	Left Middle Orbital Gyrus
60	0.011	4.92	<0.001	46	-62	46	Right Angular Gyrus
**Intensify > Permit**							
				— No results —			
**Intensify > Distance**							

24	0.003	3.72	<0.001	-18	-10	-12	Left Amygdala (ROI, transient)
**Distance > Intensify**							
1381	<0.001	7.34	<0.001	54	-54	42	Right Inferior Parietal Lobule
337	<0.001	5.98	<0.001	-52	-60	42	Left Inferior Parietal Lobule
166	<0.001	5.85	<0.001	-34	48	-4	Left Middle Orbital Gyrus
311	<0.001	5.79	<0.001	38	18	48	Right Middle Frontal Gyrus
265	0.001	5.60	<0.001	8	-64	38	Right Precuneus
274	0.001	5.45	<0.001	-2	-26	28	Left Posterior Cingulate Cortex
192	0.002	5.32	<0.001	18	30	52	Right Superior Frontal Gyrus
74	0.002	5.30	<0.001	46	46	-14	Right Inferior Frontal Gyrus

Table S4. Activation maxima during re-exposure after 10 min (sections A, B) and after 1 week (sections C, D), split into separate analyses for negative (sections A, C) and neutral (sections B, D) pictures. Abbreviations as in Table S1.

k	p_{FWE}	t	$p_{unc.}$	x	y	z	Label
**A. RE-EXPOSURE AFTER 10 MINUTES (NEGATIVE PICTURES)**							
**Regulation effect (negative pictures)**							
				— No results —			
**DistanceNegative > PermitNegative**							
				— No results —			
**PermitNegative > DistanceNegative**							
				— No results —			
**PermitNegative > IntensifyNegative**							
				— No results —			
**IntensifyNegative > PermitNegative**							
				— No results —			
**IntensifyNegative > DistanceNegative**							
				— No results —			
**DistanceNegative > IntensifyNegative**							
				— No results —			
**B. RE-EXPOSURE AFTER 10 MINUTES (NEUTRAL PICTURES)**							
k	p_{FWE}	t	$p_{unc.}$	x	y	z	Label
**Regulation effect (neutral pictures)**							
				— No results —			
**DistanceNeutral > PermitNeutral**							
				— No results —			
**PermitNeutral > DistanceNeutral**							
				— No results —			
**PermitNeutral > IntensifyNeutral**							
				— No results —			
**IntensifyNeutral > PermitNeutral**							
				— No results —			
**IntensifyNeutral > DistanceNeutral**							
				— No results —			
**DistanceNeutral > IntensifyNeutral**							
				— No results —			

C. RE-EXPOSURE AFTER 1 WEEK (NEGATIVE PICTURES)							
k	p_{FWE}	t	$p_{unc.}$	x	y	z	Label
**Regulation effect (negative pictures)**							
				— *No results* —			
**DistanceNegative > PermitNegative**							
				— *No results* —			
**PermitNegative > DistanceNegative**							
				— *No results* —			
**PermitNegative > IntensifyNegative**							
				— *No results* —			
**IntensifyNegative > PermitNegative**							
				— *No results* —			
**IntensifyNegative > DistanceNegative**							
				— *No results* —			
**DistanceNegative > IntensifyNegative**							
				— *No results* —			

D. RE-EXPOSURE AFTER 1 WEEK (NEUTRAL PICTURES)							
k	p_{FWE}	t	$p_{unc.}$	x	y	z	Label
**Regulation effect (neutral pictures)**							
				— *No results* —			
**DistanceNeutral > PermitNeutral**							
				— *No results* —			
**PermitNeutral > DistanceNeutral**							
				— *No results* —			
**PermitNeutral > IntensifyNeutral**							
40	0.003	5.43	<0.001	-40	-28	30	N/A
**IntensifyNeutral > PermitNeutral**							
				— *No results* —			
**IntensifyNeutral > DistanceNeutral**							
				— *No results* —			
**DistanceNeutral > IntensifyNeutral**							
				— *No results* —			

Table S5. Pairwise comparisons of subjective arousal ratings. Values represent p-values for pairwise t-tests after Bonferroni-Holm correction for multiple comparisons.

**Contrast**	t	p
PermitNegative > DistanceNegative	2.81	0.013
IntensifyNegative > PermitNegative	8.38	<0.001
IntensifyNegative > DistanceNegative	10.68	<0.001
PermitNeutral > DistanceNeutral	2.86	0.013
IntensifyNeutral > PermitNeutral	5.21	<0.001
IntensifyNeutral > DistanceNeutral	9.34	<0.001
DistanceNegative > DistanceNeutral	11.98	<0.001
PermitNegative > PermitNeutral	9.62	<0.001
IntensifyNegative > IntensifyNeutral	11.14	<0.001

Table S6. Pairwise comparisons of amygdala summary statistics according to different picture and regulation conditions during the stimulation phase. Values represent p-values for pairwise t-tests after Bonferroni-Holm correction for multiple comparisons.

	Transient		Sustained	
**Contrast**	left	right	left	right
PermitNegative $vs.$ DistanceNegative	0.023	0.037	0.068	0.515
PermitNegative $vs.$ IntensifyNegative	0.792	1.000	1.000	1.000
IntensifyNegative $vs.$ DistanceNegative	$<$0.001	0.001	0.033	0.319
PermitNeutral $vs.$ DistanceNeutral	0.792	1.000	0.064	0.021
PermitNeutral $vs.$ IntensifyNeutral	0.790	1.000	1.000	1.000
IntensifyNeutral $vs.$ DistanceNeutral	0.114	0.874	0.070	0.096
DistanceNeutral $vs.$ DistanceNegative	0.321	0.861	0.068	0.021
PermitNeutral $vs.$ PermitNegative	0.002	0.001	0.277	0.580
IntensifyNeutral $vs.$ IntensifyNegative	$<$0.001	$<$0.001	0.020	0.102

Table S7. Pairwise comparisons of amygdala summary statistics for negative stimuli according to different regulation conditions during the stimulation and post-stimulation phase. Values represent p-values for pairwise t-tests after Bonferroni-Holm correction for multiple comparisons.

	Transient		Sustained	
**Contrast**	left	right	left	right
PermitStim $vs.$ DistanceStim	0.036	0.057	0.054	0.368
PermitStim $vs.$ IntensifyStim	1.000	1.000	1.000	1.000
IntensifyStim $vs.$ DistanceStim	$<$0.001	0.001	0.025	0.213
PermitPostStim $vs.$ DistancePostStim	1.000	1.000	1.000	0.667
PermitPostStim $vs.$ IntensifyPostStim	1.000	1.000	1.000	1.000
IntensifyPostStim $vs.$ DistancePostStim	1.000	1.000	1.000	0.948
DistancePostStim $vs.$ DistanceStim	0.146	0.652	0.121	0.028
PermitPostStim $vs.$ PermitStim	1.000	1.000	0.001	$<$0.001
IntensifyPostStim $vs.$ IntensifyStim	0.328	0.497	0.001	0.001

Table S8. Pairwise comparisons of amygdala summary statistics according to different picture and regulation conditions during the re-exposure experiments. Values represent p-values for pairwise t-tests after Bonferroni-Holm correction for multiple comparisons.

	after 10 min		after 1 week	
**Contrast**	left	right	left	right
PermitNegative $vs.$ DistanceNegative	1.000	1.000	0.138	0.357
PermitNegative $vs.$ IntensifyNegative	1.000	1.000	1.000	1.000
IntensifyNegative $vs.$ DistanceNegative	1.000	1.000	1.000	1.000
PermitNeutral $vs.$ DistanceNeutral	1.000	1.000	1.000	1.000
PermitNeutral $vs.$ IntensifyNeutral	1.000	1.000	1.000	1.000
IntensifyNeutral $vs.$ DistanceNeutral	1.000	1.000	1.000	1.000
DistanceNeutral $vs.$ DistanceNegative	1.000	1.000	0.034	0.134
PermitNeutral $vs.$ PermitNegative	1.000	1.000	1.000	1.000
IntensifyNeutral $vs.$ IntensifyNegative	1.000	1.000	1.000	1.000
